# Prominent epigenetic and transcriptomic changes in CD4^+^ and CD8^+^ T cells during and after pregnancy in women with multiple sclerosis and controls

**DOI:** 10.1186/s12974-023-02781-2

**Published:** 2023-04-27

**Authors:** Alberto Zenere, Sandra Hellberg, Georgia Papapavlou Lingehed, Maria Svenvik, Johan Mellergård, Charlotte Dahle, Magnus Vrethem, Johanna Raffetseder, Mohsen Khademi, Tomas Olsson, Marie Blomberg, Maria C. Jenmalm, Claudio Altafini, Mika Gustafsson, Jan Ernerudh

**Affiliations:** 1 Division of Automatic Control, Department of Electrical Engineering, Linköping University, Linköping, Sweden; 2 Bioinformatics, Department of Physics, Chemistry and Biology, Linköping University, Linköping, Sweden; 3 Division of Inflammation and Infection, Department of Biomedical and Clinical Sciences, Linköping University, Linköping, Sweden; 4 Department of Obstetrics and Gynecology, Region Kalmar County, Kalmar, Sweden; 5 Department of Neurology, Linköping University, Linköping, Sweden; 6 Department of Biomedical and Clinical Sciences, Linköping University, Linköping, Sweden; 7 Department of Clinical Immunology and Transfusion Medicine, Linköping University, Linköping, Sweden; 8 Neuroimmunology Unit, Department of Clinical Neuroscience, Center for Molecular Medicine, Karolinska Institute, Karolinska University Hospital, Stockholm, Sweden; 9 Department of Obstetrics and Gynecology, Linköping University, Linköping, Sweden

**Keywords:** Multiple sclerosis, Pregnancy, CD4^+^, CD8^+^, T cells, Methylation, Transcriptomics, Networks, Modules

## Abstract

**Background:**

Multiple sclerosis (MS) is a neuroinflammatory disease in which pregnancy leads to a temporary amelioration in disease activity as indicated by the profound decrease in relapses rate during the 3rd trimester of pregnancy. CD4^+^ and CD8^+^ T cells are implicated in MS pathogenesis as being key regulators of inflammation and brain lesion formation. Although Tcells are prime candidates for the pregnancy-associated improvement of MS, the precise mechanisms are yet unclear, and in particular, a deep characterization of the epigenetic and transcriptomic events that occur in peripheral T cells during pregnancy in MS is lacking.

**Methods:**

Women with MS and healthy controls were longitudinally sampled before, during (1st, 2nd and 3rd trimesters) and after pregnancy. DNA methylation array and RNA sequencing were performed on paired CD4^+^ and CD8^+^ T cells samples. Differential analysis and network-based approaches were used to analyze the global dynamics of epigenetic and transcriptomic changes.

**Results:**

Both DNA methylation and RNA sequencing revealed a prominent regulation, mostly peaking in the 3rd trimester and reversing post-partum, thus mirroring the clinical course with improvement followed by a worsening in disease activity. This rebound pattern was found to represent a general adaptation of the maternal immune system, with only minor differences between MS and controls. By using a network-based approach, we highlighted several genes at the core of this pregnancy-induced regulation, which were found to be enriched for genes and pathways previously reported to be involved in MS. Moreover, these pathways were enriched for in vitro stimulated genes and pregnancy hormones targets.

**Conclusion:**

This study represents, to our knowledge, the first in-depth investigation of the methylation and expression changes in peripheral CD4^+^ and CD8^+^ T cells during pregnancy in MS. Our findings indicate that pregnancy induces profound changes in peripheral T cells, in both MS and healthy controls, which are associated with the modulation of inflammation and MS activity.

**Supplementary Information:**

The online version contains supplementary material available at 10.1186/s12974-023-02781-2.

## Introduction

Multiple sclerosis (MS) is an autoimmune disease of the central nervous system (CNS), to a large degree driven by T cell-mediated inflammation [1]. Despite recent advances in immunomodulatory treatments, many people with MS continue to deteriorate. To identify biomarkers for personalized treatment and to develop better treatment options, we need a better understanding of the disease-promoting and -alleviating immune mechanisms MS. Interestingly, women with MS (wwMS) show a marked decrease in disease activity during pregnancy, while this improvement is disrupted at delivery and followed by a transient worsening after pregnancy [2]. Thus, pregnancy and the period after pregnancy (post-partum) provides a useful model to assess the dynamics of immune regulation in MS. Of note, pregnancy is one of the most profound suppressors of disease activity, with a 70–80% reduction in relapse rate in the 3rd trimester [2, 3], thereby exceeding the effects of many currently available treatments. Indeed, pregnancy induces profound and timely tuned immune adaptations in the maternal immune system to ensure tolerance of the semi-foreign fetus [4]. Thereby these changes, which involve both innate and adaptive immune responses, help to avoid inflammation-mediated pregnancy complications [4, 5]. The phenomenon of pregnancy-associated immunomodulation is plausibly a result of immune-endocrine changes involving increase in levels of pregnancy hormones, such as progesterone and estrogen, which gradually rise and peak in the 3rd trimester of pregnancy [6]. Notably, the period following delivery is associated with a temporary worsening of disease activity, coinciding with a rapid decline in pregnancy hormone levels [7]**.** A better understanding of how the modulation of the maternal immune system affects MS could provide insights into central disease mechanisms as well as facilitate the discovery of new treatment strategies.

T cells are key regulators of inflammation, and in accordance [8], they are central in the regulation of tolerance during pregnancy [9] as well as in the regulation of MS pathology [1]. Extensive evidence supports a crucial role for peripherally activated autoreactive T cells in MS, where CD4^+^ and CD8^+^ T cells are implicated in both initiating and propagating the disease [10, 11]. Also, we recently observed that pregnancy can affect autoimmune disease-associated methylation changes in T cells [12], which further supports a pregnancy-specific modulation of T cells of relevance for MS. Several studies have previously shown the involvement of T cells in MS during pregnancy [13–18], which has shed light on the pregnancy-associated modulation, although the precise effects of pregnancy on CD4^+^ and CD8^+^ T cells remain relatively unknown. Further, it is unclear if the changes induced during pregnancy are MS-specific or a general consequence of pregnancy.

High-throughput approaches are well-suited tools to characterize the molecular events associated with the dynamic immune changes occurring during pregnancy. Analysis of pregnancy-induced transcriptomic [19, 20] and epigenetic changes [21] in the 3rd trimester compared to post-partum (PP) in wwMS and healthy controls (HC), has led to the identification of molecular signatures of potential immune-regulatory markers. However, no studies have specifically utilized omics approaches in CD4^+^ and CD8^+^ T cells to study dynamic changes during pregnancy. In particular, combining mRNA expression profiling of T cells, which provides a snapshot of the current state of the cells, together with the epigenetic regulation could provide a more comprehensive view of the underlying molecular events of relevance for the T cell modulation in MS during pregnancy.

In the present study, in-depth characterization using genome-wide methylation status and mRNA expression of paired samples from circulating CD4^+^ and CD8^+^ T cells, longitudinally collected throughout and after pregnancy, showed that pregnancy induced large epigenetic and transcriptomic changes in both wwMS and HC. Interestingly, the most prominent changes were observed in the 3rd trimester, which rebounded post-partum, thus mirroring the effect of pregnancy on the disease activity in MS. Using a network-based approach to capture the most highly interconnected genes, we identified CD4^+^ and CD8^+^ rebound pregnancy modules that were overlapping between the two omics and disclosed central genes and pathways involved in T cell regulation and differentiation. Our findings highlight a systemic pregnancy-induced modulation of T cells, coinciding with the temporary improvement and worsening of MS, which could be associated with the modulation of inflammation and MS disease activity. The enrichment of MS-associated genes and MS-relevant pathways further supports the importance of T cell regulation in MS and during pregnancy.

## Results

### Pregnancy induces genome-wide epigenetic and transcriptomic changes

**Fig. 1:**
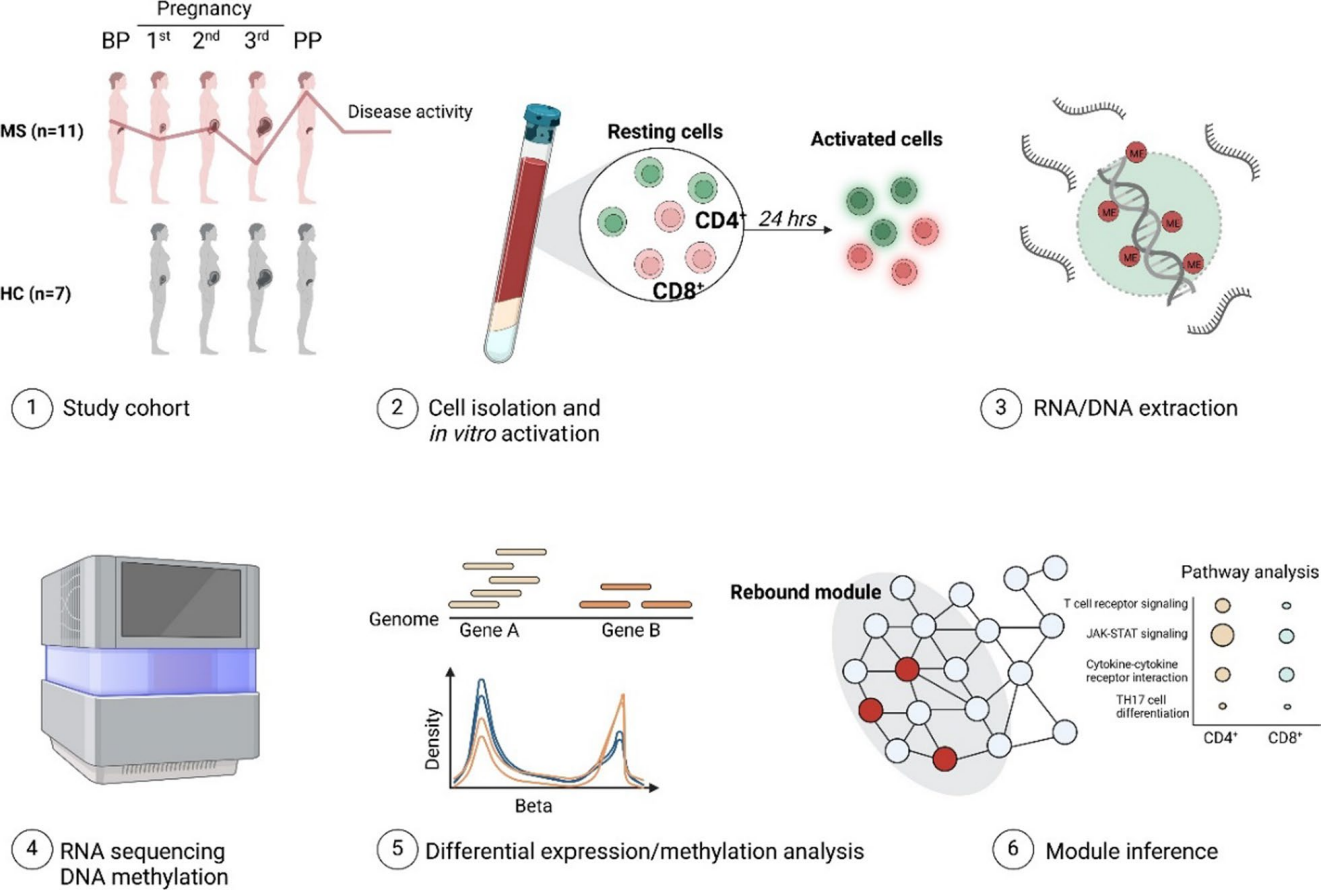
Overview of the study. CD4^+^ and CD8^+^ T cells were isolated from women with MS and healthy controls (HC) that were longitudinally sampled throughout and after pregnancy (1st, 2nd, 3rd trimester and post-partum). For MS, samples were also included before pregnancy. RNA and DNA were extracted from resting and in vitro activated cells. RNA-sequencing was performed to investigate transcriptomic changes and genome-wide profiling of DNA methylation was performed using the Illumina Infinium DNA Methylation EPIC Array. Differentially expressed genes (DEGs) and differentially methylated genes (DMGs) were used to construct modules using the module inference tool DIAMOnD. *BP* before pregnancy, *HC* healthy controls, *MS* multiple sclerosis, *PP* post-partum

**Fig. 2:**
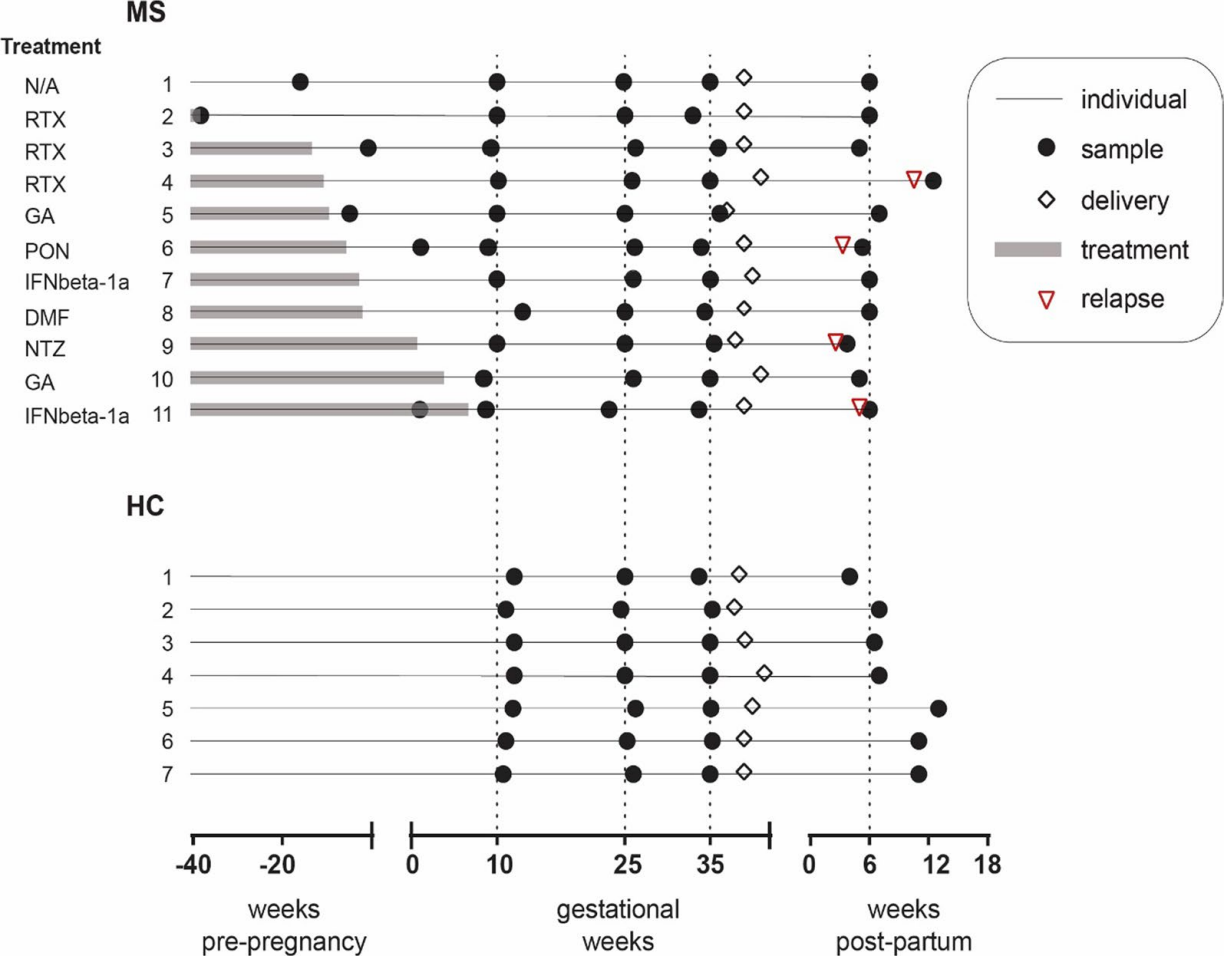
Study cohort characteristics. Schematic overview of the pregnant women with MS (*n*=11) and healthy controls (HC, *n*=7) included in the study. Shown in the figure as black circles is the sampling point (in gestational weeks) during the 1st, 2nd, and 3rd trimesters of pregnancy, and the sampling point post-partum or before pregnancy (in weeks, the latter available only for some of the women with MS). The gestational week of delivery (white rhombus), and, for the women with MS, the time since the latest treatment (grey line) and the type of treatment are also depicted. Women with MS were recruited at Karolinska University Hospital, Stockholm, Sweden (*n*=7) and Linköping University Hospital, Linköping, Sweden (*n*=4). All healthy pregnant controls (*n*=7) were recruited at Kalmar County Hospital, Kalmar, Sweden. DMF; dimethyl fumarate (*n*=1), GA; glatiramer acetate (*n*=2), IFNbeta-1a; interferon beta-1a (*n*=2), NTZ; natalizumab (*n*=1), PON; ponesimod (*n*=1), RTX; rituximab (*n*=3), untreated (*n*=1)

**Fig. 3:**
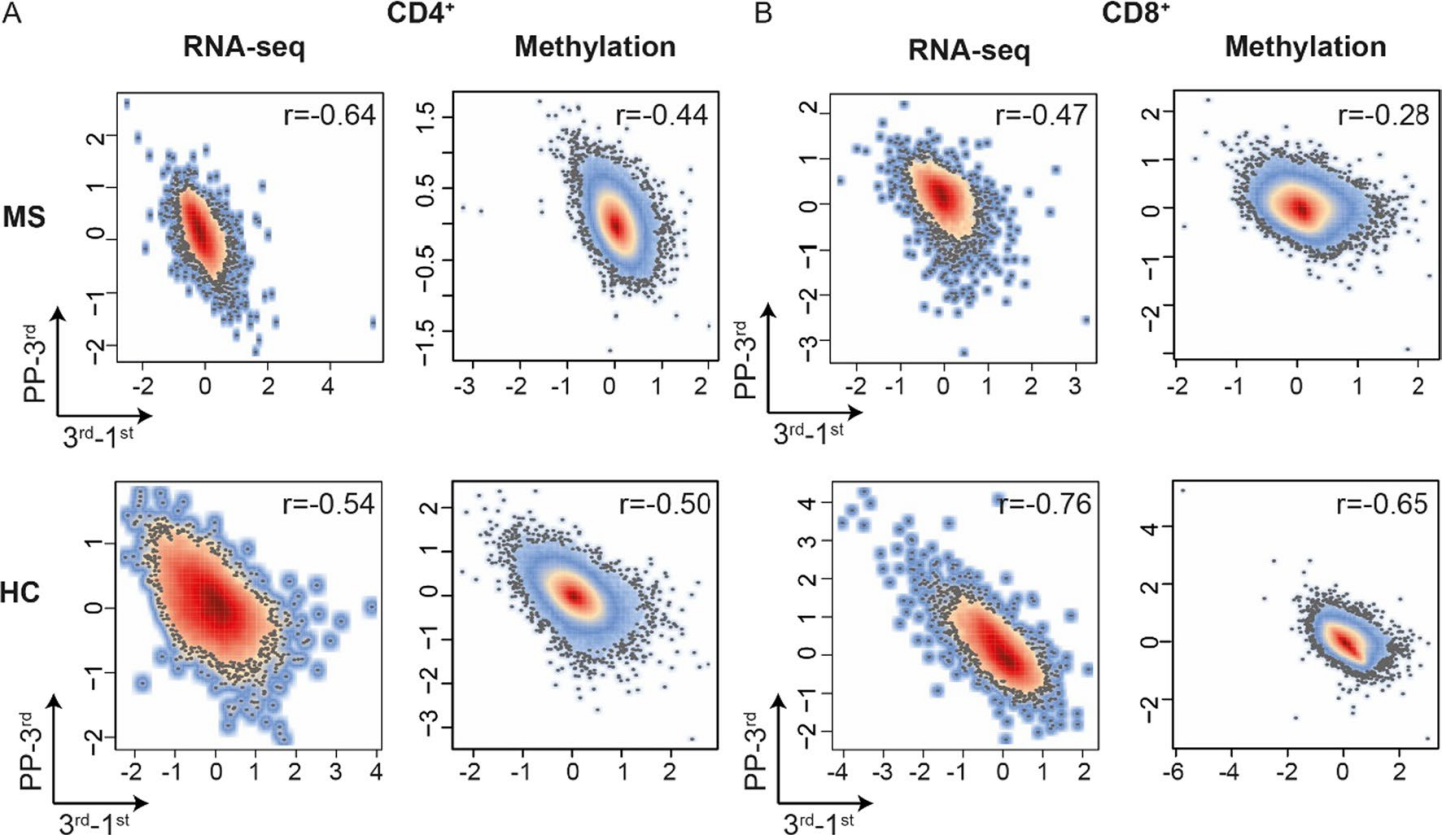
Pregnancy induces genome-wide epigenetic and transcriptomic changes in CD4^+^ and CD8^+^ T cells. DNA and RNA extracted from CD4^+^ and CD8^+^ T cells from women with MS and healthy controls (HC) were analyzed during pregnancy by RNA-seq and Illumina Infinium DNA Methylation EPIC Array for DNA methylation. The Pearson correlation coefficients between gene counts (for RNA-seq) and the beta values of all detected CpGs (for methylation) for the comparisons 3rd-1st trimester and PP-3rd trimester for resting **A** CD4^+^ cells and **B** CD8^+^ cells in women with MS (upper panel) and HC (lower panel) are shown. Pearson’s correlation *r* is shown in the individual graphs for each comparison. All correlations had a *p* < 2.2 x 10^−16^. *HC* healthy controls, *MS* multiple sclerosis, *PP* post-partum

In this explorative study, wwMS and HC were longitudinally blood sampled during and after pregnancy to capture the dynamic effects of pregnancy on MS (Table 1, Figs. 1, 2). An overview and the general design of the study is presented in Fig. 1 and Additional file 1: Figs. S1, S2. Resting and in vitro activated isolated CD4^+^ and CD8^+^ T cells were analyzed by RNA sequencing (RNA-seq) and DNA methylation array (only resting cells) to delineate the transcriptomic and epigenetic changes underlying the disease modulation that occurs during pregnancy (see Additional file 1: Fig. S3 and Additional file 2: Table S1 for a detailed description of the samples). To get an initial global understanding of the changes induced by pregnancy, we investigated the changes across all genes (CD4^+^ *n* = 13,440 genes; CD8^+^ *n* = 13,448 genes) and CpGs (*n* = 740,552 for both cell types). The changes occurring throughout pregnancy correlated well in MS and HC (Pearson’s *r* 0.48–0.77, all correlations had *p* < 2.2 × 10^–16^), at both expression and methylation level (Additional file 1: Fig. S4). We thus concluded that pregnancy induces consistent genome-wide changes in both groups. Conversely, a significant negative correlation was noted between the changes induced during pregnancy and after pregnancy, which was generally more pronounced around the 3rd trimester, as compared to the 2nd (Fig. 3A, B and Additional file 1: Fig. S5). This pattern was further investigated by differential analysis. However, the number of features (i.e., genes or CpGs) with a false discovery rate (FDR)-corrected *p*-value ≤ 0.05 varied drastically depending on cell type, disease and omic (Additional file 3: Table S2, Additional file 4: Table S3, Additional file 5: Table S5). Therefore, to investigate the effect of pregnancy on all groups in a consistent manner, the less stringent requirement of nominal statistical significance (*p* ≤ 0.05) was chosen as criterion to identify differentially expressed genes (DEGs) and differentially methylated probes (DMPs). During pregnancy, the highest number of DEGs/DMPs was generally observed in the 3rd trimester (3rd-1st, Additional file 1: Fig. S6). Further, these changes generally showed the highest overlap with the DEGs/DMPs post-partum (PP-3rd; Additional file 1: Fig. S6), again highlighting the 3rd trimester as a central time point for maximal regulation during pregnancy. Taken together, we observed the most marked changes during the 3rd trimester and post-partum, in agreement with the effect of pregnancy on the disease activity in MS [2]. We therefore decided to focus the subsequent analyses on these two timelines, i.e., changes in 1st versus 3rd trimesters and 3rd trimester versus post-partum.

**Table 1:** Study cohort characteristics

	MS (*n* = 11)	HC (*n* = 7)
*Samples*		
Pre-pregnancy, *n*	6	N/A
1st trimester, *n*; gw median (range)	11; 10.0 (8.0–13.0)	7; 11.9 (10.7–12.0)
2nd trimester, *n*; gw median (range)	11; 25.0 (23.1–26.3)	7; 25.0 (24.6–26.3)
3rd trimester, *n*; gw median (range)	11; 35.0 (33.0–36.1)	7; 35.0 (33.7–35.3)
Post-partum, *n*; week median (range)	11; 6.0 (3.7–7.0)	7; 6.9 (4.3–12.9)
*Subject* *characteristics*		
Age (years), mean ± SD	31.8 ± 2.1	27.7 ± 3.4
BMI, mean ± SD	23.9 ± 3.8	22.7 ± 3.1
*Current* *pregnancy*		
Gw at delivery, mean ± SD	40.1 ± 0.9	39.3 ± 1.2
Fetal sex	5 males/ 6 females	3 males/ 4 females
Mode of delivery		
Vaginal delivery, *n*	10	7
Cesarean section, *n*	1	0
*Pregnancy* *history* (mean ± SD)		
Parity	0.6 ± 0.8	0.4 ± 0.8
Previous miscarriages	0.3 ± 0.9	0.1 ± 0.4
Previous live births	0.6 ± 0.8	0.4 ± 0.8
*Disease* *parameters*		
Disease duration, mean ± SD	7.4 ± 6.2	N/A
Disease severity		
EDSS at inclusion, median (range)	0.5 (0–2.5)*	N/A
EDSS post-partum, median (range)	0.5 (0–2.0) +	N/A
*Treatment*		
Treatment washout (weeks)		
From pre-P sampling, median (range)	8.0 (0–20.9)	N/A
From 1st trimester sampling, median (range)	14.9 (2.0–48.3)	N/A

*BMI* body mass index kg/m^2^, *EDSS* Expanded Disability Status Score, *gw* gestational week, *HC* healthy controls, *MS* multiple sclerosis, *SD* standard deviation

*Data on EDSS were missing for 1 individual, + data on EDSS were missing for 5 individuals

### The changes in CD4^+^ and CD8^+^ T cells during pregnancy rebound post-partum

**Fig. 4:**
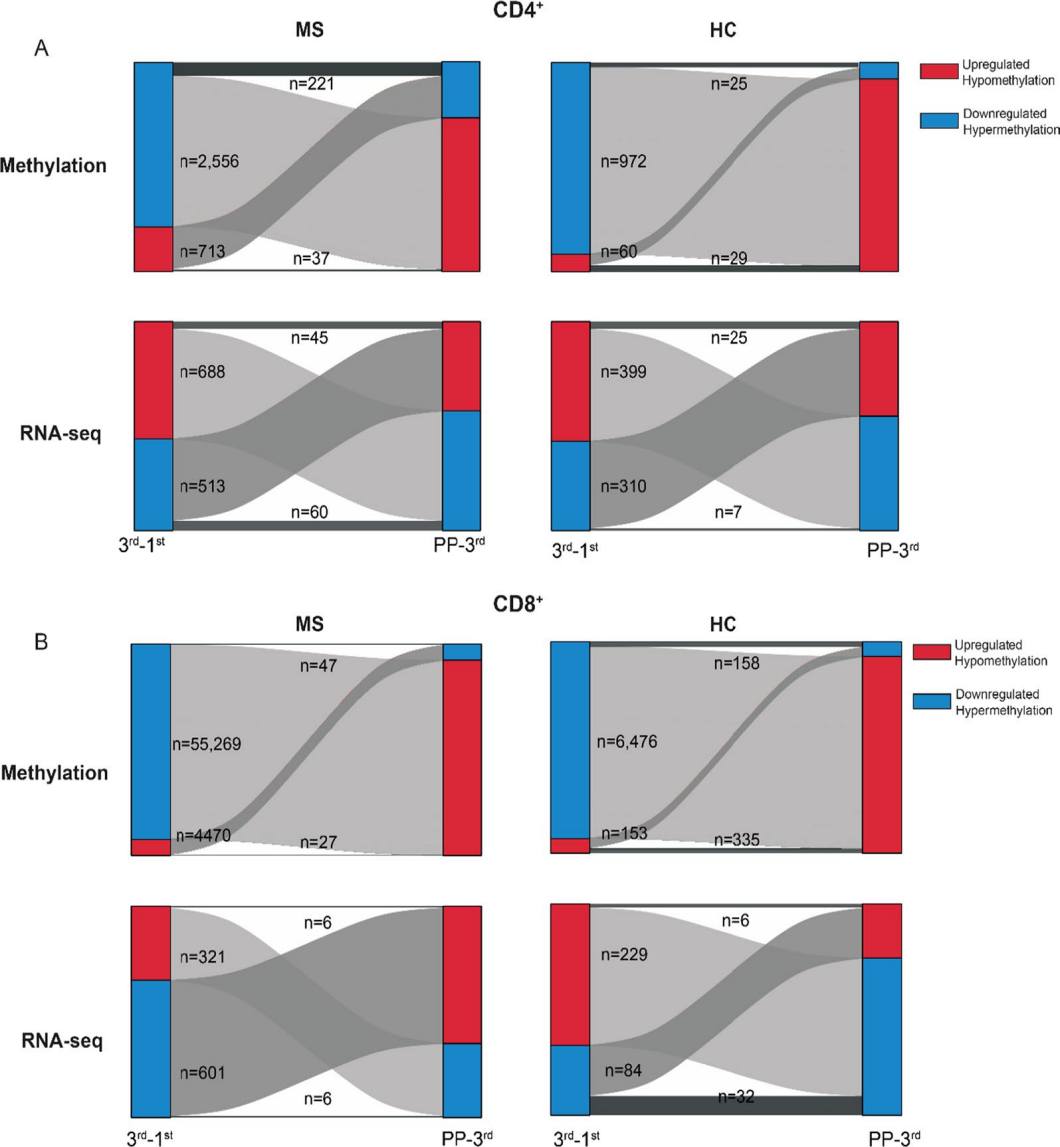
Differential analysis in CD4^+^ and CD8^+^ T cells reveals a post-partum rebound. Alluvial plots for **A** CD4^+^ and **B** CD8^+^ cells in both pregnant women with MS and pregnant healthy controls (HC) showing all CpGs (methylation) and all genes (RNA-seq) that were either differentially expressed (nominal *p*≤0.05) or differentially methylated (nominal *p*≤0.05 and |Δβ|>0.05) during (3rd-1st) or after pregnancy (PP-3rd). The genes and CpGs are grouped based on their direction during and after pregnancy (red=hypomethylation or upregulated, blue= hypermethylation or downregulated). The number of genes and CpGs in each corresponding region are depicted in the figure. *HC* healthy controls, *MS* multiple sclerosis

In both MS and HC, pregnancy was dominated by hypermethylation in both CD4^+^ and CD8^+^ T cells, whereas post-partum was characterized by hypomethylation (Fig. 4A, B). On the other hand, at the transcriptomic level, there was a more equal distribution of up- and down-regulated genes (Fig. 4A, B). In line with the genome-wide negative correlations between the changes induced during and after pregnancy, differential analysis confirmed that many of the changes observed in the 3rd trimester were reversed post-partum (Fig. 4A, B). Indeed, there was a significant overlap between the DEGs/DMPs in the 3rd trimester and post-partum (Additional file 1: Fig. S7). Strikingly, for all these overlapping DEGs/DMPs, expression and methylation changed direction post-partum, i.e., upregulated genes (respectively, hypomethylated CpGs) in the 3rd trimester became downregulated (respectively, hypermethylated) after pregnancy and, similarly, downregulated genes (hypermethylated CpGs) became upregulated (hypomethylated); these DEGs/DMPs will be hereon referred to as rebound DEGs/DMPs. The presence of a rebound in DNA methylation and gene expression was further confirmed by considering the samples collected before pregnancy (BP; only available for wwMS) as the baseline. Indeed, differential analysis identified few DMPs and DEGs between BP and PP, relative to the comparisons between BP and the 1st, 2nd and 3rd trimesters (Additional file 1: Fig. S8). However, it should be noted that for most of the probes (> 97%) and genes (> 90%) the measurements at post-partum did not reach the original levels before pregnancy (Additional file 1: Fig. S8), indicating that the observed rebound is not complete (i.e., the values at post-partum are not equal to those before pregnancy).

### Pregnancy induces similar changes irrespective of disease or not

**Fig. 5:**
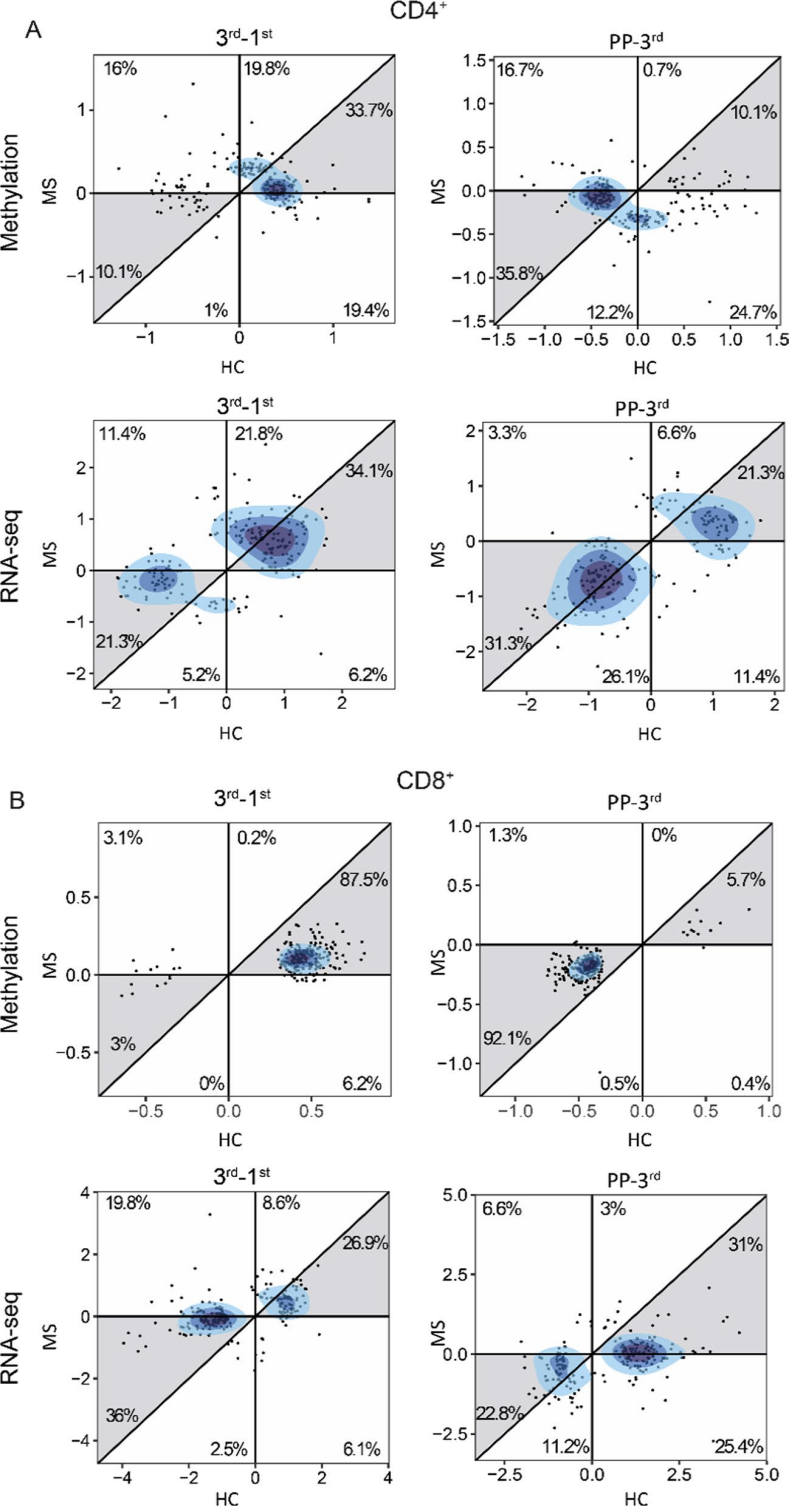
Pregnancy induces similar changes irrespective of disease. Comparison of the differentially methylated CpGs and differentially expressed genes during (3rd-1st) and after (PP-3rd) pregnancy in women with MS and healthy controls (HC) in **A** CD4^+^ and **B** CD8^+^. The axes indicate the change of methylation and expression during and after pregnancy as measured by the log_2_ of the limma coefficients β_d,3rd_ - β_d,1st_ and β_d,PP_ - β_d,3rd_, respectively, where the disease group d (MS or HC) is specified in the axis labels. The percentages of the dots in each area are shown. The colors are based on the density of the dots where darker color represents a higher density. *HC* healthy controls, *MS* multiple sclerosis

Given that the temporary improvement of disease activity is most pronounced during the 3rd trimester, we hypothesized that the identified rebound genes and CpGs could be involved in mediating these pregnancy-induced effects. Interestingly, a significant overlap was found between the rebound DEGs and DMPs in MS and HC (CD4^+^ OR = 7.9 (methylation), 43.0 (RNA-seq) and *p* = 0.03, < 2.2 × 10^–16^; CD8^+^ OR = 15.6, 22.9 and *p* < 2.2 × 10^–16^, 2 × 10^–10^). Moreover, by analyzing the directionality in the rebound DEGs/DMPs, we observed that most of them changed in the same direction in both MS and HC (Fig. 5A, B). This was particularly evident for the CD8^+^ DMPs, where > 90% showed the same pattern in both MS and HC (Fig. 5B). Further, not only did MS and HC resemble each other when considering DNA methylation and gene expression separately, but also the regulatory patterns between the two omics were highly similar. More precisely, the regulatory role of each CpG was measured as the Spearman correlation coefficient with the corresponding gene and a significant overlap was observed between CpG–gene pairs characterized by similar correlations in MS and HC, for both CD4^+^ and CD8^+^ T cells (Additional file 1: Fig. S9).

Since the changes of most rebound DEGs/DMPs shared the same directionality in MS and HC, ranging from 60 to 98% in overlap, we continued our analysis on these genes/probes, hereon referred to as shared rebound DEGs/DMPs, comprising a total of 160 and 114 DEGs for CD4^+^ and CD8^+^, respectively, and 126 and 2161 DMPs. In particular, the shared rebound DMPs were associated to 80 and 551 genes in CD4^+^ and CD8^+^, respectively, which we denoted as shared rebound DMGs.

The implication of the shared rebound DEGs/DMGs in MS was investigated by considering a list of MS-associated genes obtained by combining the database DisGeNET [22] and the latest GWAS in MS [23]. Of note, the rebound DEGs/DMGs that were MS-specific, i.e., only rebounded in MS, were not significantly enriched in MS-associated genes (data not shown), whereas the shared rebound DEGs/DMGs were significantly enriched (OR = 1.6–2.5, *p* ≤ 0.05 as determined by Fisher’s exact test, Additional file 1: Fig. S10), highlighting that the changes relevant for MS are affected and modulated by pregnancy itself, and not specific to the disease. Interestingly, the shared rebound DEGs/DMGs were also significantly enriched for genes affected during our in vitro T cell activation (CD4^+^ OR = 3.2, *p* = 2.1 × 10^–13^, CD8^+^ OR = 2.5, *p* = 6.7 × 10^–6^), suggesting their potential importance in T cell regulation (Additional file 1: Fig. S10). Surprisingly, even though for example the JAK–STAT signaling pathway was significantly enriched in CD4^+^ T cells in both omics (Additional file 1: Fig. S10), there was little to no overlap between the DEGs and DMGs in either CD4^+^ or CD8^+^ T cells; more precisely, the overlap was only 2 out of 106/98 (DEGs/DMGs) in CD4^+^ HC, 48 out of 158/8,850 in CD8^+^ HC, 0 out of 84/45 in CD4^+^ MS, and 0 out of 49/258 in CD8^+^ MS.

### ﻿﻿Using a network-based modular approach reveals common immune-mediated multi-omics processes during pregnancy

**Fig. 6:**
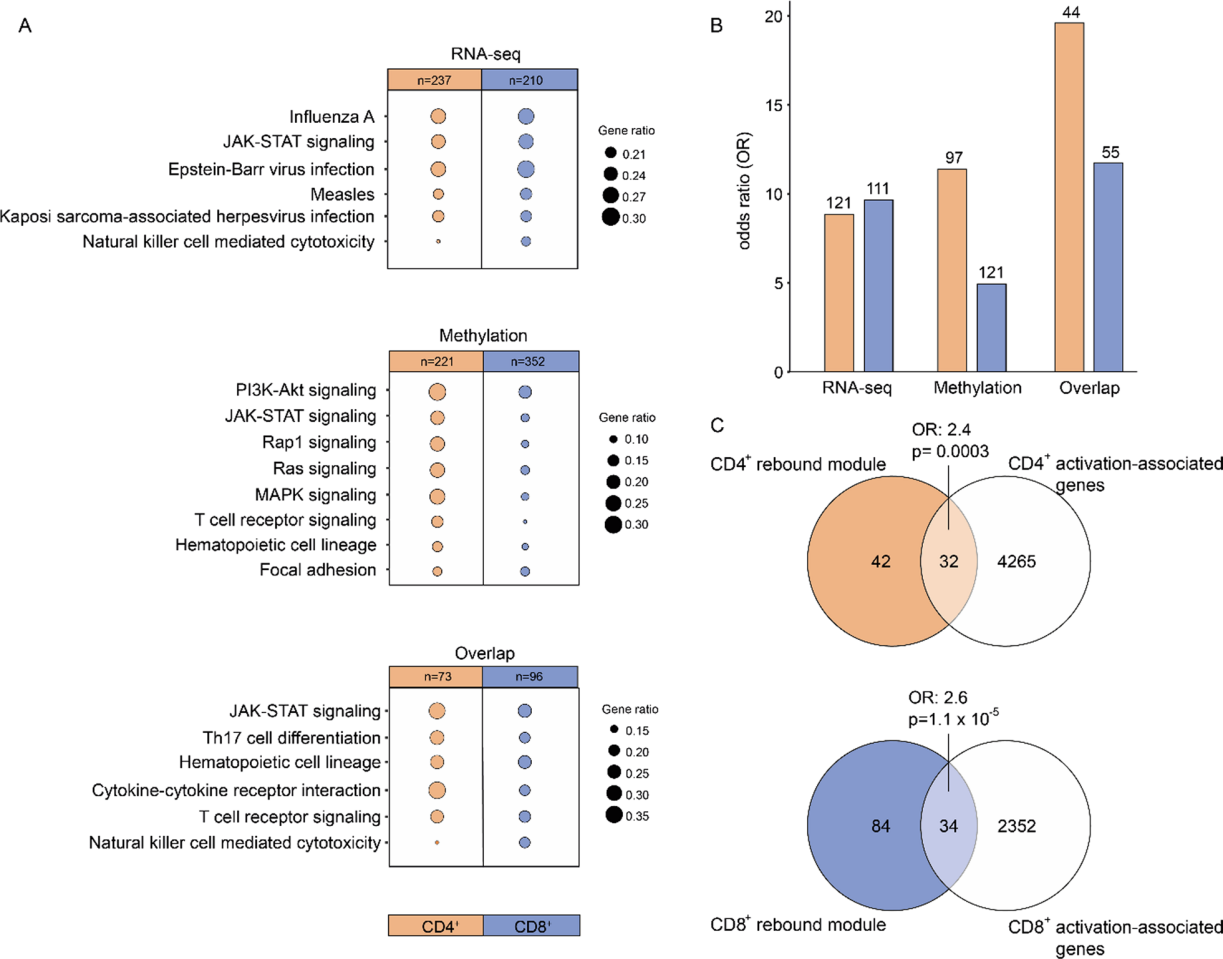
A network-based modular approach reveals common immune-mediated *multi-omics* processes during pregnancy. Overlapping differentially expressed genes (DEGs) and differentially methylated probes (DMPs) between the 3rd trimester and post-partum were characterized by changes in expression and methylation of opposite direction in these two time points in both women with MS and healthy controls (HC) and were thus termed shared rebound DEGs/DMPs. Modules were inferred from these shared rebound DEGs/DMPs using DIAMOnD and the PPI network (threshold >700). **A** KEGG pathway analysis of the module genes derived from RNA-seq, methylation data or the overlap between the modules derived from the two omics. Shown are the top 5 pathways, in terms of adjusted *p*-value, for at least one of the two groups (CD4^+^ and CD8^+^). All the pathways have an adjusted *p*-value ≤ 0.05; *n* is the total number of genes contained in the pathways of each column and gene ratio indicates the number of genes in a pathway divided by the total number of module genes. **B** Enrichment of the module genes for MS-associated genes retrieved from GWAS-derived MS genes and DisGeNET (all comparisons had *p* < 2.2e−16) in transcriptomics and methylomics of CD4^+^ and CD8^+^ T cells. The number of overlapping genes is shown above each bar. **C** The overlap between the genes derived from shared genes derived from the RNA-seq and methylation modules, termed rebound module, for each cell type, respectively, and the genes associated to the activated cells. Enrichment and overlaps were computed using Fisher’s exact test, *p* ≤ 0.05 was considered statistically significant.

**Fig. 7:**
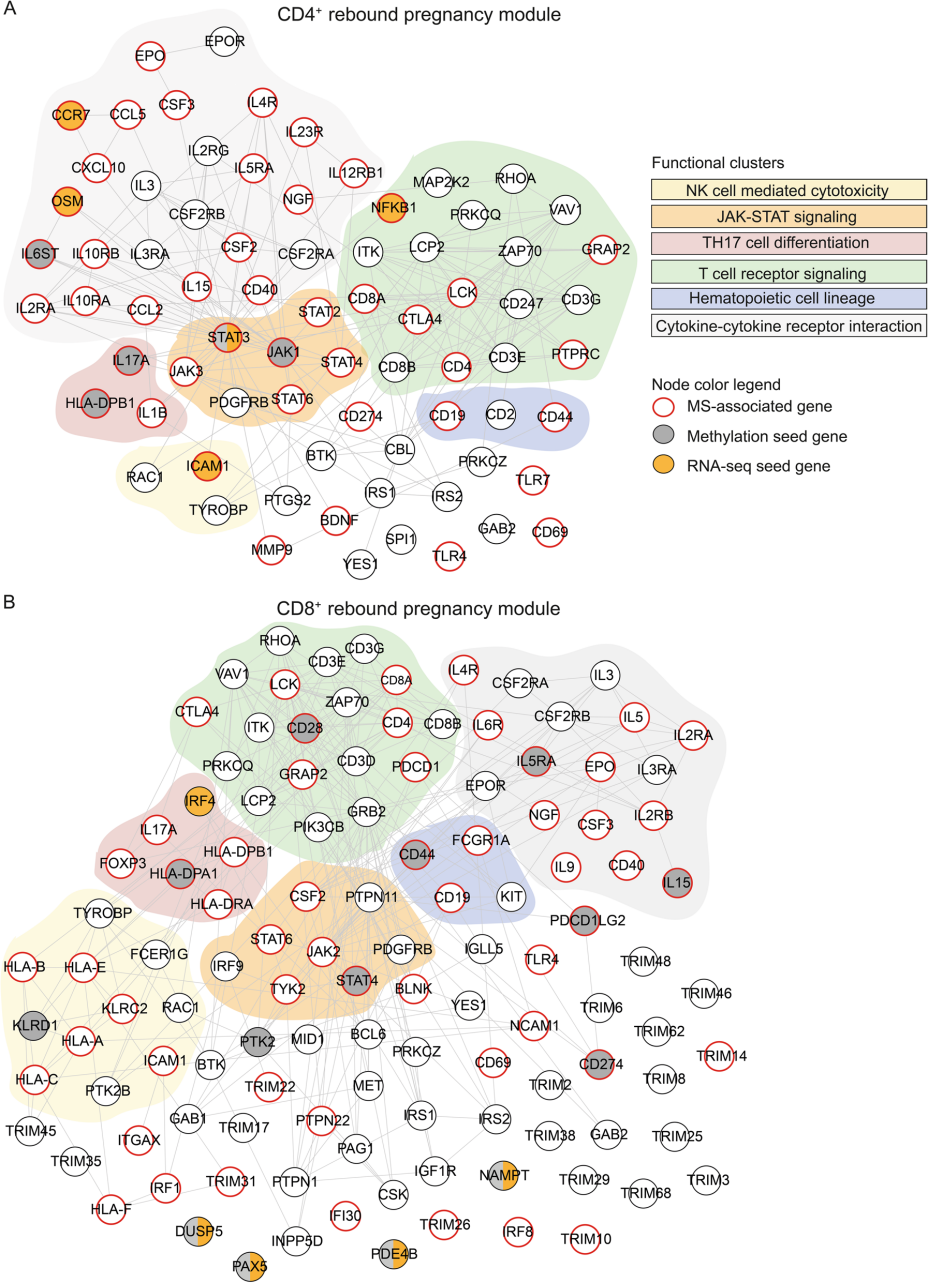
CD4^+^ and CD8^+^ rebound pregnancy modules. **A**, **B** Graphical illustration of the rebound pregnancy modules for CD4^+^ and CD8^+^ T cells, respectively. The rebound pregnancy modules were derived by overlapping the module genes from the RNA-seq and methylation module from each cell type. The module inference method DIAMOnD was used to construct the original modules for each omic and cell type separately. Nodes represent genes and the connecting edges show the protein–protein interactions. The networks were created based on the protein–protein interaction networks from STRINGdb (threshold > 950). Functional clustering of the genes was performed by KEGG pathway analysis. For illustrative purposes, each gene was assigned to only one pathway. Some genes were annotated to several similar pathways and the complete list of pathways, and their annotated genes can be found in Additional file 8: Table S7. *MS* multiple sclerosis

The observation that some pathways appeared in both cell types (CD4^+^ and CD8^+^ T cells) and omics (methylation and transcription) suggested that the shared rebound DEGs/DMGs, despite the modest overlap between the actual genes, could be involved in similar biological processes. To further explore this possibility, we employed a network-based modular approach to identify more functionally related genes. In particular, we applied DIAMOnD [24] from the module inference package MODifieR [25] on the shared rebound DEGs/DMGs. DIAMOnD is based on the observation that genes involved in complex diseases are characterized by a high level of connectivity in the protein–protein interaction (PPI) network, compared to random proteins, thereby forming modules consisting of highly interconnected genes [24]. Using DIAMOnD, we identified four different pregnancy modules, i.e., one per cell type and omic, ranging from 259 to 590 genes per module (Additional file 7: Table S6). The pregnancy modules showed an overall enrichment for immune-related pathways associated with T cell signaling and differentiation, such as T cell receptor signaling and JAK–STAT signaling (Fig. 6A, Additional file 8: Table S7), emphasizing T cell regulation and differentiation as central processes that are affected during pregnancy. Furthermore, the pregnancy modules were all highly enriched in MS-associated genes and genes associated with our experimentally induced T cell activation (Fig. 6B, C). We indeed found a significant overlap between the shared rebound methylation and RNA-seq modules for both cell types (CD4^+^ OR = 42.7, CD8^+^ OR = 40.9, *p* < 2.2 × 10^–16^), highlighting a functional relationship between the shared rebound DEGs and DMGs. This resulted in 74 common genes for CD4^+^ and 118 common genes for CD8^+^ (Additional file 7: Table S6), hereon referred to as CD4^+^ and CD8^+^ rebound pregnancy modules, respectively (Fig. 7A, B). These module genes were also enriched for pathways related to T cell differentiation and signaling (Fig. 6A) and were even more highly enriched for MS-associated genes as compared to the separate pregnancy modules (CD4^+^ OR = 19.6, *p* < 2.2 × 10^–16^; CD8^+^ OR = 11.7, *p* < 2.2 × 10^–16^; Fig. 6B). To further validate the biological relevance of the identified modules, we used the data from a previous published independent study, where we identified a set of 1992 genes that were significantly affected by progesterone (P4) in CD4^+^ T cells [26]. P4 is one of the major pregnancy hormones, with pronounced anti-inflammatory properties, whose levels during and after gestation coincide with the temporary improvement and worsening of disease activity in MS. It has therefore been suggested to be one of the main drivers of the pregnancy-induced modulation of MS [6]. Indeed, we found that the rebound genes, and even more markedly the rebound pregnancy modules, were significantly enriched for these P4-associated genes (CD4^+^ OR = 3.3, *p* < 5 × 10^–4^; CD8^+^ OR = 3.2, *p* < 7 × 10^–4^; Additional file 1: Fig. S11). In summary, by using a network-based approach we identified a set of genes that were shared across both omics (and cell types) and reflected the dynamics of disease-associated changes as well as central T cell-related processes.

## Discussion

Despite the evidence that T cells play a central role in MS [10], few studies have investigated how these cells are affected by the course of pregnancy, a potent suppressor of inflammation and disease activity. Using longitudinally collected blood samples during and after pregnancy, we examined the genome-wide DNA methylation and gene expression patterns in peripheral CD4^+^ and CD8^+^ T cells from wwMS and HC. Pregnancy induced prominent dynamic changes in both methylation and gene expression patterns, converging during the 3rd trimester, where most changes were common between MS and HC. The majority of pregnancy-induced changes rebounded post-partum, in accordance with the pregnancy-induced modulation of disease activity. By using a network-based modular approach, we identified pregnancy modules based on the rebound genes and their most highly interconnected genes. These modules were significantly enriched in MS-associated genes and disclosed central genes and pathways involved in T cell regulation such as JAK–STAT and T cell receptor signaling. Interestingly, the modules overlapped significantly between the omics for each cell type, respectively, highlighting changes that are regulated both at methylation and expression level.

Studies on systemic effects induced by pregnancy emphasize that the changes from pre-pregnancy to the 3rd trimester are reversed post-partum [13, 19, 27, 28], coinciding with the temporary improvement during the 3rd trimester and worsening of the disease post-partum [2]. The notion of the 3rd trimester representing a pivotal time point in immune regulation during pregnancy is further emphasized by the increased risk of certain infections at this time point [29]. In accordance, we found that the 3rd trimester and the post-partum period were characterized by the largest changes in the differential analysis, mostly revealing that the induced changes during the 3rd trimester were reversed post-partum. In particular, we found that the progression of pregnancy was associated with an increasingly hypermethylated state, in agreement with previous findings [12]. On the other hand, we noticed a more equal distribution of up- and downregulated genes at the transcriptomic level, which could partly be explained by the dual demands on the maternal immune system during pregnancy, preserving effective immunity while maintaining fetal tolerance [4].

Although T cells are central in driving inflammatory responses in MS, previous studies on pregnancy have reported diverging results on how T cells are modulated during this time of transient tolerance, which might reflect differences in study design and methodology [14, 15, 21]. We found dynamic alterations in the epigenetic and transcriptomic profiles of both CD4^+^ and CD8^+^ T cells during the course of pregnancy, alterations that were subsequently reversed post-partum. Importantly, we found that the observed changes rather represent a general adaption of the maternal immune system during pregnancy, where only a small number of genes were influenced specifically in the presence of MS. Strikingly, the small number of genes that were altered in MS but not in HC were not enriched in MS-associated genes. These small differences are in agreement with other studies that have investigated changes in the immune system during pregnancy in MS [13, 14, 28], which is also in line with similar observations in RA [30] where pregnancy was shown to have a stronger impact than the presence of disease. Taken together, our results suggest that the decrease in inflammatory activity in MS observed during pregnancy is likely due to the general immune adaptation that takes place during this time, thus not an MS-specific phenomenon but rather a bonus effect of general pregnancy-induced changes.

T cell responses have generally been suggested to shift towards a more anti-inflammatory status during pregnancy to protect the semi-allogenic fetus, which could explain the temporary modulation of T cell-mediated diseases, although the precise mechanisms remain unclear [31, 32]. Single-cell transcriptomic analysis of peripheral blood mononuclear cells (PBMCs) throughout healthy pregnancy showed that pathways related to T cell activation and regulation were dampened during pregnancy [33], highlighting the need to suppress potentially alloreactive T cells. Our modular approach revealed central genes and pathways involved in T cell regulation and differentiation such as JAK–STAT signaling, T cell receptor signaling and Th17 cell differentiation, which were shared across both omics. We found that pregnancy affected many MS-associated genes, including for example *CXCL10,* *IL17A*, *STAT3* and the T cell activation marker *CD69*. Simultaneously, the rebound modules were also significantly enriched in genes associated with our in vitro T cell activation, which is consistent with previous studies showing that MS is associated with a dysregulation in response to activation in CD4^+^ T cells [34, 35]. Taken together, pregnancy seemingly influences genes and pathways that are highly relevant in driving inflammatory processes in MS, which could play a role in the pregnancy-induced modulation of MS by potentially affecting autoreactive T cells [13].

The pregnancy hormones P4 and estrogen have been considered main drivers behind the modulation of the maternal immune system during pregnancy [6]. The changes in hormonal concentrations during and after pregnancy, coinciding with the temporary improvement and worsening of MS, highlight a potential role of sex hormones in the pregnancy-induced alterations of MS. We and others have previously shown that P4 significantly dampens T cell activation [26, 36–39], whereas estrogens seem to exert both immune-activating and immune-dampening effects [37, 40, 41]. Indeed, estrogen treatment in humans has not shown convincing results [6, 42] while clinical trials investigating progesterone/progestins are largely missing. Interestingly, we found that the pregnancy modules were significantly enriched for genes known to be affected by P4 [26], indicating important circuits of interactions between P4, T cells and inflammatory activity in MS. Further studies looking into the potential effect of P4 and progestins as add-on treatment in MS are highly needed.

While the relatively small sample size of our study is a potential limitation, our longitudinal study design with multiple samples taken from the same individual helps to reduce the variance in the data, thereby increasing the signal-to-noise ratio. Still, the huge number of genes (> 13,000) and methylation sites (850,000 CpGs) could explain the lack of probes and genes surviving FDR correction in the initial analyses. It was thus important to find ways to strengthen the biological signal and limit the number of false positives. Therefore, we applied a twofold analysis approach. Firstly, we focused solely on nominally significant changes that were corroborated by multiple time points. The fact that these DEGs and DMPs showed a high degree of similarity in MS and HC, both in terms of overlap and direction, was taken as an indication that they represented a robust set of core changes related to pregnancy. Secondly, module inference was used to enhance the signal of the input genes and aid our understanding of multi-omics data. In fact, although there was little overlap at the gene level between the omics, the original DEGs and DMGs pointed to similar pathways. By using our network-based approach, we were able to identify core sets of genes that were shown to significantly overlap between RNA-seq and methylation data.

Another potential limitation is that we have only investigated changes related to CD4 and CD8 T cells. T cells are implicated as main drivers in MS [10, 23], supported both by animal studies and genome-wide association studies, and are highly relevant in the immune regulation during pregnancy [9]. However, there are other cell types, such as B cells and monocytes, involved in the MS pathogenesis that would be highly relevant to study in the context of disease-modulation during pregnancy that should be considered in future studies.

This study represents, to our knowledge, the first global methylation and expression analysis that evaluates the temporal changes induced in peripheral CD4^+^ and CD8^+^ T cells during pregnancy in MS. Our findings emphasize pregnancy as a potent modulator of central immune cells in the MS pathogenesis and underscore the need for further studies investigating treatment strategies that can mimic the pregnancy milieu.

## Materials and methods

An overview of the study is shown in Fig. 1 and Additional file 1: Figs. S1 and S2.

### Study population

The present study utilized blood samples from 11 pregnant wwMS and 7 pregnant HC, who were enrolled in the Pregnancy-MS study, a prospective longitudinal cohort study performed at four centers in Sweden. In the study, women with relapsing–remitting MS planning to get pregnant (before pregnancy (BP)) or wwMS and HC in the first trimester of pregnancy were included and followed longitudinally during pregnancy (1st, 2nd, and 3rd trimesters) and 6 weeks post-partum. Blood samples were collected from a total of 54 wwMS and 30 HC with singleton pregnancies. wwMS were recruited at Karolinska University Hospital, Solna, Linköping University Hospital, Linköping, and Ryhov County Hospital, Jönköping, and controls were recruited at Region Kalmar County, Kalmar. All sites followed an identical sampling procedure. Eligible for the study were women of Caucasian ethnicity, aged 18–45 years. Women with immune-associated or other severe diseases (in addition to MS in the MS group), as well as women pregnant after/by assisted reproductive technique/in vitro fertilization (IVF) or a history of previous obstetric complications were not considered for the study. The study was performed in accordance with the Helsinki Declaration’s ethical principles for medical research and was approved by the Regional ethical review board in Linköping (2012/402-31). All participants signed informed consent.

Out of the 54 wwMS enrolled in the study, three failed to get pregnant, one had a miscarriage in gestational week (gw) 13, one had an elective abortion in gw 14, and 11 dropped out of the study for personal reasons after the first sampling occasion. From the remaining 38 wwMS, the selection of the 11 who were finally included in the present study, was based on the availability of complete sample sets, the treatment washout period before pregnancy (the ones with the longest washout were prioritized), and the absence of relapses and/or MS-related treatments during pregnancy. Nevertheless, 4 of the included women experienced a relapse post-partum and in addition, there was one case of hypothyroidism. Out of the 30 HC recruited in the Pregnancy-MS study, one woman had a miscarriage in gw 12, ten dropped out of the study after the initial sampling, and eight had one or more missing samples during pregnancy and/or post-partum resulting in 18 women being eligible to be included in the present study. From them, the seven women were randomly chosen among the ones with no pregnancy complications, however there was one case of depression (not pharmacologically treated). There was a significant difference in the age between the groups (MS: (mean ± SD) 31.8 ± 2.1, HC: 27.7 ± 3.4, *p*: 0.0054, unpaired t test). No difference was noted for body mass index (BMI). Further, there were no differences in fetal sex, mode of delivery, previous miscarriages and previous live births between MS and HC (determined by Fisher’s exact test). The sampling was performed in the 1st trimester (for MS: gestational week (gw) median (range) 10.0 (8.0–13.0), HC: gw 11.9 (10.7–12.0)), in the 2nd trimester (MS: gw 25.0 (23.1–26.3), HC: gw 25.0 (24.6–26.3)), in the 3rd trimester (MS: gw 35.0 (33.0–36.1), HC: gw 35.0 (33.7–35.3)), and after delivery (MS: week 6.0 (3.7–7.0), HC: week 6.9 (4.3–12.9)). The sampling time differed significantly between MS and HC for the 1st trimester (*p*: 0.0046, unpaired t test). The characteristics of the cohort are shown in Table 1 and Fig. 2.

### Isolation of peripheral blood mononuclear cells

Venous blood was collected in Vacutainer® CPT™ with sodium citrate (BD Bioscience, Franklin Lakes, NJ, USA) and centrifuged for 15 min, 1500 × *g* at room temperature (RT). After removal of the plasma, the cell suspension was washed twice in Dulbecco’s phosphate-buffered saline (DPBS; Thermo Fisher Scientific, Waltham, MA, USA). The cells were resuspended in 20% dimethyl sulfoxide (Sigma-Aldrich, Saint Louise, MO, USA) with 80% heat-inactivated fetal bovine serum (FBS, Sigma-Aldrich) and placed in a CoolCell® (Corning®; Corning, NY, USA) for at least 4 h before being transferred and stored in liquid nitrogen until further use.

### Positive selection of CD4^+^ and CD8^+^ T cells

The PBMCs were transferred from liquid nitrogen and thawed in a 37 °C water bath and washed twice for 10 min, 400 × *g* in RT in pre-heated (at 37 °C) Iscove’s modified Dulbecco’s medium (IMDM; Invitrogen, Carlsbad, CA, USA) supplemented with l-glutamine (292 mg/mL; Sigma-Aldrich), MEM non-essential amino acids 100X (10 ml/L; Gibco®, Thermo Fisher Scientific), penicillin–streptomycin (5000 U/mL; Lonza™ BioWhittaker™, Thermo Fisher Scientific), sodium bicarbonate (3.024 g/L, Sigma-Aldrich) and 10% FBS (HyClone™; Thermo Fisher Scientific). The cells were resuspended in Hank’s Balanced Salt Solution (HBSS; Thermo Fisher Scientific) and filtered through a pre-separation filter (30 µm; Miltenyi﻿﻿﻿﻿ Bio tec, Bergisch Gladbach, North Rhine-Westphalia, Germany).

The cells were counted in a Bürker chamber (Hecht Assistant®, Sondheim vor der Rhön, Germany) and the viability was assessed by Trypan Blue (Thermo Fisher Scientific) showing an average viability of 86%.

CD8^+^ and CD4^+^ T cells were separated by immunomagnetic positive selection according to the instructions provided by the manufacturer using MS columns and miniMACS separators (Miltenyi Biotec). The CD8^+^ cells were isolated first and the CD4^+^ cells were subsequently isolated from the CD8^−^ fraction. A small portion of the cells was processed for flow cytometry. The remainder of cells were either (1) resuspended in RLT Plus Buffer (Qiagen, Hilden, Germany) using syringe and needle to lyse and homogenize the cells and transferred to − 70 °C until RNA/DNA extraction or (2) processed for in vitro stimulation (see below). The average viability before culture was 87% for CD4^+^ and 88% for CD8^+^ cells and the purity was > 88% in CD4^+^ (mean 96%) and > 86% (mean 94%) in CD8^+^, based on the flow cytometry analysis (for gating strategy see Additional file 1: Figs. S12 and S13).

### In vitro stimulation

Twenty-four well plates (Corning) were pre-coated overnight at 4 °C with 0.25 µg/mL anti-CD3 (low endotoxin clone UCHT1) and anti-CD28 (low endotoxin clone YTH913.12) antibodies (both from Bio-Rad AbD Serotec Limited, Hercules, CA, USA). The plates were washed three times in PBS (Medicago, Uppsala, Sweden). The CD8^+^ and CD4^+^ T cells were cultured in IMDM + 5%FBS (Thermo Fisher Scientific) for 24 h at 37 °C and 5% CO_2_ at a density of 2 million cells/mL. After culture, a proportion of the cells were processed for flow cytometry and the rest were homogenized and lysed in RLT Plus Buffer (Qiagen) and stored at − 70 °C until extraction. The proportion of live cells (evaluated by LIVE/DEAD™ Fixable Aqua Dead Cell Stain, see details below) was > 68% for the activated CD4^+^ cells and > 72% for the activated CD8^+^ cells. The average expression of CD69 after activation in all samples (as determined by flow cytometry) was for CD4^+^ MS: 5.4% (standard deviation ± 1.8) and HC: 9.5% (± 4.6); CD8^+^ MS: 15.4% (± 7.2) and HC: 25.6% (± 6.5) (Additional file 1: Fig. S14).

### Flow cytometry

Before and after the in vitro stimulation, the cells were analyzed by flow cytometry. The CD8^+^ and CD4^+^ T cells were resuspended in LIVE/DEAD™ Fixable Aqua Dead Cell Stain (Invitrogen), diluted according to the instructions provided by the company, and labeled with mouse anti-human CD3-PE (UCHT-1), CD4-PeCy7 (SK3) or CD8-PeCy7 (SK1), CD45RA-V450 (HI100) and CD69-APCCy7 (FN50; all purchased from BD Biosciences, San Jose, CA, USA) for 15 min in the dark at RT. The cells were resuspended in PBS + 0.1% FBS prior to analysis. Ten thousand cells were collected and analyzed using FACS Canto II (BD Biosciences) and Kaluza flow cytometry software version 2.1 (Beckman Coulter, Fullerton, CA, USA). For information regarding the gating strategy, see Additional file 1: Figs. S12 and S13.

### Extraction of DNA and RNA

Total DNA and RNA were isolated using the Quick-DNA/RNA™ Microprep Plus Kit (Zymo Research, Irvine, CA, USA) according to the manufacturer’s instructions. The RNA and DNA concentrations were determined using a Qubit™ 3.0 fluorometer (Thermo Fisher Scientific) with the Qubit™ RNA BR Assay kit for RNA and Qubit™ dsDNA BR Assay Kit for DNA (both from Thermo Fisher Scientific). The integrity of the extracted RNA was evaluated using the Agilent RNA 6000 Nano Kit (Agilent Technologies, Santa Clara, CA, USA) on an Agilent 2100 Bioanalyzer (Agilent Technologies). The median RNA Integrity (RIN) was 8.8 (range 7.3–10). For two samples, RIN values were not measurable but since there was a sufficient amount of RNA, the samples were included and processed for library preparation and subsequent sequencing.

### DNA methylation

DNA from 154 samples (from 78 resting CD4^+^ and 76 resting CD8^+^ T cells, Additional file 2: Table S1 and Additional file 1: Fig. S3) was sent to the SNP&SEQ-technology platform at SciLifeLab (Uppsala University, Uppsala, Sweden). Only 6 samples were available BP in MS and due to insufficient amount of material, samples were also missing for two time points in CD8^+^ in MS (Additional file 2: Table S1 and Additional file 1: Fig. S3). Bisulfite conversion was performed using the EZ DNA Methylation™ Kit (Zymo Research) with 250 ng of DNA per sample as input. The bisulfite converted DNA was eluted in 15 μl Elution Buffer according to the manufacturer´s protocol, evaporated to a volume of < 4 μl, and used for methylation analysis using Infinium Human MethylationEPIC BeadChip array (Illumina) covering 850,000 methylation sites across the genome.

### RNA sequencing

Samples (resting and activated cells, *n* = 233) were sent to the National Genomics Infrastructure in Stockholm (SciLifeLab) for RNA sequencing. Library preparations were carried out on an Agilent NGS Bravo workstation (Agilent Technologies) in 96-well plates following the instructions provided for the Illumina TruSeq Stranded mRNA kit from Illumina (Illumina). Briefly, mRNA was purified from 200 ng of total RNA through selective binding on poly dT-coated beads and fragmented using divalent cations under elevated temperature. cDNA was synthesized from the resulting fragments by adding SuperScript II Reverse Transcriptase (Thermo Fisher Scientific). This step was followed by bead clean-up with the AMPure XP solution (Thermo Fisher Scientific) to selectively retain fragments of desired lengths. The cDNA was then subjected to 3’ adenylation, followed by adapter ligation to the 3’ adenylated end of the fragment. The fragments with ligated adapters were cleaned on AMPure XP beads to remove non-ligated adapters and were amplified by PCR. The PCR products were purified by binding to AMPure XP beads, washed with 80% ethanol, and eluted in elution buffer (Qiagen, Hilden, Germany). The quality of the adapter-ligated libraries was checked on the BioAnalyzer or LabChip® GX/HT DNA High Sensitivity Kit (PerkinElmer, Waltham, MA, USA), and their concentration was determined by Quant-iT (Thermo Fisher Scientific). Because of technical errors at the sequencing facility, 34 samples failed library preparation. The libraries with concentrations above 20 nM were normalized and pooled, and the concentration of the final pools was estimated by qPCR. The pool was sequenced on the NovaSeq S6000 (Illumina) on 3 lanes of the S4-300 (v1.5) flowcell. After sequencing, 2 samples were excluded due to insufficient sequencing depth. A total of 197 samples were successfully sequenced, passing FASTQC and with a high enough sequencing depth (average 67 × 10^6^ million reads per sample) and were included in subsequent analyses, which resulted in samples from 65 resting CD4^+^ cells, 51 activated CD4^+^ cells, 61 resting CD8^+^ cells and 20 activated CD8^+^ cells (Additional file 2: Table S1 and Additional file 1: Fig. S3). The difference in sample availability resulted in an uneven distribution of some samples across time points and groups, particularly for the activated samples where limited material was available for performing the T-cell activation assay (see Additional file 2: Table S1 and Additional file 1: Fig. S3 for a detailed description of sequenced samples). The samples were demultiplexed and pre-processing was carried out using the nf-core/rnaseq3.0 pipeline (https://github.com/nf-core/rnaseq). Briefly, quality control was performed using FASTQC (version 0.11.9; Babraham Institute https://bioinformatics.babraham.ac.uk) and trimming of low-quality reads and adapter contamination was done using TrimGalore! (version 0.6.6; Babraham Institute). Paired-end reads were aligned and mapped to the Ensemble human reference genome GRCh38 (Genome Reference Consortium Human Build 38) using STAR [43] (version 2.6.1d). Gene count was quantified using Salmon [44] (version 1.4.0).

### Bioinformatics analysis

Data obtained from the DNA methylation and RNA sequencing were analyzed using the programming language R (version. 4.2.1); the code is available at https://github.com/albertozenere/GraMS.

### Pre-processing of methylation and RNA sequencing data

Pre-processing of the data generated by Infinium Human MethylationEPIC BeadChip (Illumina) was performed using the package ChAMP [45] (version 2.22.0). It included filtering out probes: (i) with a detection *p*-value < 0.01, (ii) with a bead count less than 3, (iii) with no GC start, (iv) close to a SNP, following the list in [46]. The number of probes that survived the filtering was 740,552. The data were normalized using the Beta-Mixture Quantile dilation method [47] and batch correction was carried out using ComBat [48], where the slide effect was removed while the biological signal coming from cell type, time and disease was preserved. When applicable, CpGs were mapped to genes using the annotation provided by Illumina. For the RNA-seq, batch correction was performed with ComBat-seq [49], contained in the package sva (version 3.44.0), where the library batch effect was corrected and the signal associated to cell type, time, disease and our in vitro activation was retained. Normalization was carried out with edgeR (version 3.38.1) using the TMM method [50]. Genes with low counts, i.e., with count-per-million below 10 in 70% of the samples, were filtered out. In total, 13,440 genes for CD4^+^ and 13,448 genes for CD8^+^ cells were retained (for both resting and activated samples).

### Differential analysis

Methylation and RNA-seq data were modeled using the R package limma [51] (version 3.52.1). Regarding the methylation data, differential expression analysis was performed on the M-values, which have been shown to be more appropriate for differential analysis [52]. The measurements of each CpG and gene were fitted to account for the effect of disease group (MS or HC), time (BP, 1st, 2nd, 3rd trimester and post-partum), age, the proportion of memory cells, and cell viability. The data were fitted by the linear model:

$$y= {\beta }_{0}+ {\beta }_{d,t}*s+ {\beta }_{a}*a+{\beta }_{m}*m+{\beta }_{c}*c,$$

where y indicates the measurements of a CpG or gene, ${\beta }_{0}$ is the intercept, $d$ is the disease group, $t$ denotes time, $s$ is a term that includes both the disease group and time, *a* represents the age, *m* the proportion of memory cells and *c* the cell viability. ${\beta }_{d,t}, {\beta }_{a}, {\beta }_{m},{\beta }_{c}$ are the coefficients associated with the disease group and time, age, the proportion of memory cells and cell viability, respectively. Although there were no statistically significant differences in the proportion of naïve and memory cells across time or between groups (Additional file 1: Fig. S15), the observed differences in methylation patterns between naïve and memory T cells [53, 54] are a potential confounder, and therefore the proportion of memory cells was also included as a covariate in the model for both the methylation and RNA-seq analysis. The effects of disease and time were grouped under the same term to capture disease-specific changes over time. The individual effect (samples from the same donor tend to be correlated) was modeled as a random effect using the function *duplicateCorrelation*. The heteroscedasticity of RNA-seq data was accommodated using the function *voom* [55]. For each disease group, the coefficients ${\beta }_{d,t}$ were used as a measure of the effect of time on the data. Moderated t-statistics and corresponding *p*-values were computed using the limma function *eBayes.* Differentially methylated probes (DMPs) were defined as CpGs with a nominally significant *p*-value (*p* ≤ 0.05) and an average absolute change in beta value greater than 0.05, where CpGs with a positive value were considered hypermethylated and negative as hypomethylated. Each gene associated to at least one DMP was considered differentially methylated (DMG). Genes were defined as differentially expressed if the corresponding *p*-value was nominally significant (*p* ≤ 0.05). Since there were very few differences between BP and the 1st trimester during pregnancy in MS (< 2% of DEGs/DMPs; Additional file 1: Fig. S8), and since BP samples were not available in the HC group, the BP samples were omitted in the remaining analyses and the 1st trimester samples were used as the baseline to evaluate the changes associated with pregnancy in both groups. DMPs and DEGs in the 3rd trimester and post-partum were visualized using the package ggalluvial (version 0.12.3).

### Genes affected by in vitro T cell activation

A separate differential analysis was performed to identify genes that were statistically affected by activation at the mRNA level, as measured by the proportion of cells expressing the activation marker CD69 as determined by flow cytometry. Notably, the samples collected from HC were characterized by higher levels of activation compared to their MS counterparts (Additional file 1: Fig. S14), with statistically significant differences around the 2nd trimester and post-partum in CD4^+^ cells (Additional file 1: Fig. S14). For this reason, the level of activation was added as covariate in the model to ensure it would not confound the results. Differential analysis was performed to identify genes that were significantly (FDR-adjusted *p*-values ≤ 0.05) affected by the state of activation (i.e., resting or activated).

### Module inference

The protein–protein interaction (PPI) network was downloaded from the STRING database [56] (version 11) and filtered to contain only interactions with a high confidence defined by a combined score of at least 700. To identify a set of functionally related genes affected by pregnancy, the common DMGs and DEGs between the 3rd trimester and post-partum, referred to as rebound DEGs/DMGs, were used as input (also denoted as seed genes) for module inference, which was performed with DIAMoND [24], using the R package MODifieR [25] (version 0.1.3). For each omic and cell type, a maximum number of 200 genes were added to the initial set of genes to form the CD4^+^ and CD8^+^ modules for RNA-seq and methylation. As the RNA-seq and methylation pregnancy modules of each cell type showed a significant degree of overlap, we selected the common genes to form CD4^+^ and CD8^+^ rebound pregnancy modules, which were visualized using Cytoscape [57] (version 3.8.2). For visualization, a threshold of score ≥ 950 for the PPI was used.

### Enrichment and pathway analysis

The biological importance of both seed and module genes was tested in multiple ways. Pathway enrichment analysis, based on KEGG terms, was performed using the function *compareCluster* from clusterProfiler [58] (version 3.16.1). The results were corrected for the background (i.e., all measured genes with the inclusion of the PPI network genes when testing module genes), and the pathways with an adjusted *p*-value ≤ 0.05 were considered significant. To assess the enrichment of MS-associated genes we combined DisGeNET [22] (*n* = 1117 genes) with a list of GWAS-derived genes. For the GWAS-associated genes, we used 26,033 MS-associated SNPs derived from the latest GWAS in MS [23] (*p* < 10^–6^) and mapped them to the closest genes (tssRegion -3000 to 3000) using ChIPseeker [59] (version 1.31.4), which resulted in a total of 573 genes. Combined genes from both GWAS and DisGeNET resulted in a list of a total of 1550 MS-associated genes. Fisher’s exact test was used to assess the enrichment of MS-associated genes in seed and module genes. Lastly, we evaluated the enrichment for a recently reported list of P4-associated genes [26] using Fisher’s exact test.

## Supplementary Information

**Additional file 1: Figure S1.** Overview of the experimental set up. **Figure S2.** Overview of the analysis workflow from initial raw data to differential analysis and module inference. Rebound DEGs/DMPs were defined as the overlap between the differentially expressed genes/differentially methylated probes identified in the 3rd trimesterand post-partumsimultaneously, calculated for MS and HC separately. Genes associated with at least one rebound DMP were denoted rebound DMGs. Rebound DEGs/DMGs in common between MS and HC were termed shared rebound DEGs/DMGs. These genes served as input for creating RNA-seq and methylation modules for each cell type separately. The RNA-seq and methylation modules were overlapped to create one CD4^+^ rebound pregnancy module and one CD8^+^ rebound pregnancy module. DEGs and DMPs with a nominal p-value ≤0.05were included for analysis. DEG, differentially expressed gene; DMG, differentially methylated gene; DMP, differentially methylated probe; MMD, mean methylation difference. **Figure S3.** Overview of the samples used for RNA sequencing and DNA methylation from women with MS and healthy controls. A number of samples were excluded for the RNA-sequencing due to technical issues at the sequencing facility. Two samples were also excluded after sequencing due to insufficient sequencing depth. The number of samples is stated as the number of resting/activatedor resting cells alonein the two lower panels. HC, healthy controls; MS, Multiple sclerosis. **Figure S4.** DNA and RNA extracted from CD4^+^ and CD8^+^ T cells from women with MS and healthy controlswere analyzed by RNA-seq and Infinium Methylation EPIC 850 K for DNA methylation. Shown are the correlation between gene countsand the beta values of all detected CpGsfor the comparisons 3rd-2nd trimester and 2nd -1st trimester for resting CD4^+^ cells and CD8^+^ cells in women with MS and HC. Pearson’s correlation r is shown in the individual graphs for each comparison. All correlations had a p < 2.2 x 10^-16^. HC, healthy controls; MS, multiple sclerosis. **Figure S5.** DNA and RNA extracted from CD4^+^ and CD8^+^ T cells from women with MS and healthy controlswere analyzed by RNA-seq and Infinium MethylationEPIC 850K for DNA methylation. The correlation between gene countsand the beta values of all detected CpGsbetween PP-2nd trimester and 2nd-1st trimester for resting CD4^+^ cells and CD8^+^ cells in women with MS and HC. Pearson’s correlation r is shown in the individual graphs for each comparison. All correlations had a p < 2.2 x 10^-16^. HC, healthy controls; MS, multiple sclerosis, PP; post-partum. **Figure S6.** Number of nominally differentially expressed genesand differentially methylated CpGsduring pregnancy and post-partum comparing 2nd-1st, 3rd-2nd , 3rd-1st, PP-3rd and PP-2nd.Number of overlapping genes and CpGs during pregnancy and after pregnancy, i.e., PP-2nd compared to 2nd-1st and PP-3rd compared to 3rd-2nd and 3rd-1st trimesters. DEGs; differentially expressed genes, DMPs; differentially methylated probes, HC; healthy controls, MS; multiple sclerosis, PP; post-partum. **Figure S7.** Venn diagrams showing the overlap between the nominally differentially expressed genes or differentially methylated CpGsduringand after pregnancyin women with MS and healthy controls. Fisher’s exact test was used to calculate the enrichment of the overlaps. Odds ratios are shown, and all overlaps had a p < 10^-16^. HC; healthy controls, MS; multiple sclerosis, OR; odds ratio, PP; post-partum. **Figure S8.** Differential analysis between the samples collected before pregnancy and the remaining time points.Number of DMPs and DEGsfor each comparison.Number of probes and genes that are hypermethylated/hypomethylatedin each comparison. BP, before pregnancy; PP, post-partum; DEG, differentially expressed gene; DMP, differentially methylated probe. **Figure S9.** Regulatory patterns between DNA methylation and gene expression are conserved between MS and HC. The regulation exerted by each CpG was measured by the Spearman correlation coefficient with the respective gene, as annotated by Illumina. Correlations were computed independently in CD4^+^ and CD8^+^, for MS and HC. The correlations obtained in each of the four groups were divided into deciles based on their absolute values. The overlap between CpG–gene pairs that belong to the same decile in both MS and HC was carried out using Fisher’s exact test. **p < 0.01, ****p < 0.0001, Δ p < 10^-16^. **Figure S10.** The CD4^+^ and CD8^+^ rebound genes were derived by overlappingthe differentially expressed genesfrom 3rd-1st trimester and PP-3rd in both women with MS and healthy controlsandGenesfor the same comparisons. These genes were later used to infer modules.Enrichment of MS-associated genes based on GWAS-derived MS genes and DisGeNET; p denotes p-value. Overlap between the genes significantly affected by activation, performed only on the genes derived from the RNA-seq analysis.KEGG pathway enrichment. Shown are the top 5 pathwaysof both groups. Number of genesis shown above. Enrichment was calculated using Fisher’s exact test and p ≤ 0.05 was considered statistically significant. DEGs; differentially expressed genes, DMPs; differentially methylated probes, MS; multiple sclerosis. Shown are the top 5 pathwaysof both groups, with adjusted p-value ≤ 0.05. **Figure S11.** Enrichment of P4-associated genes. The rebound seed genes, the resulting module genes for each omic and the shared genes derived from combining the modules for both RNA-seq and methylation for each cell typewere tested for enrichment of P4-associated genes using Fisher’s exact test. The P4-associated geneswere derived from Hellberg et al., Front Immunol. ns; non-significant, P4; progesterone. *p < 0.05, ****p < 0.0001. **Figure S12.** Flow cytometry characterization of resting and activated CD4^+^ T cells. Flow cytometry gating strategies to assess purity, viability, and activation status in resting and activated CD4^+^ T cells. Viable cells were identified as Aqua- and further gated based on forwardand sidescatter to further characterize CD4^+^ cells. The cut-off value for CD69 expression was based on the expression in the resting cells. Definition of naïve and memory T cells was based on the contour of the CD45RA+and CD45RA-populations. Viability, purity of the isolated cells, activation leveland proportion of naïve and memory CD4^+^ T cells were evaluated on resting cells on day 0. Activated cells were analyzed for viability, CD69 expressionand proportion of naïve and memory cells. The figure shows one representative sample. **Figure S13.** Flow cytometry characterization of resting and activated CD8^+^ T cells. Flow cytometry gating strategies to assess purity, viability, and activation status in resting and activated CD8^+^ T cells. Viable cells were identified as Aqua- and further gated based on forwardand sidescatter and to characterize CD8+ cells. The cut-off value for CD69 expression was based on the expression in the resting cells. Definition of naïve and memory was based on the contour of the CD45RA^+^ and CD45RA^–^ populations. Viability, purity of the isolated cells, activation leveland proportion of naïve and memory cells was evaluated on resting cells on D0. Activated cells were analyzed for viability, CD69 expressionand proportion of naïve and memory cells. The figure shows one representative sample. **Figure S14.** CD69 expression in resting and activated CD4^+^ and CD8^+^ T cells.Proportion of CD69^+^ cells among CD4^+^ or CD8^+^ resting and activated T cells combining all time points within women with MS and healthy controls. CD69 expression in activated CD4^+^ and CD8^+^ cells before pregnancy, 1st, 2nd and 3rd trimester and post-partum. No activated cells were available before pregnancy for the healthy controls. The number of activated samples differ from the number of resting samples as not all samples had enough material to perform the T-cell activation assay, resulting in fewer activated samples. Statistical differences were determined using an unpaired t test between MS and HC within each time point, respectively. BP, before pregnancy; HC, healthy controls; MS, Multiple sclerosis; PP, post-partum; trim, trimester. **p < 0.01, ***p < 0.001, ****p < 0.0001. **Figure S15.** Proportions of naïveand memoryin resting CD4^+^ and CD8^+^ cells from women with MS and healthy controlsbefore, during and after pregnancy. MS and HC were also combined to compare differences over time irrespective of disease. There were no statistical differences within each group over time, between groups over time or at the same time point. Statistical differences were determined using one-way ANOVA. No statistically significant differences were found. BP; before pregnancy, HC, healthy controls; MS, Multiple sclerosis, PP; post-partum.

**Additional file 2: Table S1.** Overview of samples used for RNA sequencing and DNA methylation.

**Additional file 3: Table. S2.** Results of differential analysis on methylation data from healthy controls.

**Additional file 4: Table. S3.** Results of differential analysis on methylation data from multiple sclerosispatients.

**Additional file 5: Table S4.** Results of differential analysis on RNA-seq data from multiple sclerosispatients.

**Additional file 6: Table. S5.** Results of differential analysis on RNA-seq data from multiple sclerosispatients.

**Additional file 7: Table S6.** Genes identified by DIAMOnD from the module inference tool MODIfieR.

**Additional file 8: Table S7.** KEGG pathway enrichment analysis of seed and module genes.

## Data Availability

The datasets supporting the conclusions of this article are available in the ArrayExpress repository, accession numbers E-MTAB-12250 (DNA methylation) and E-MTAB-12260 (RNA-seq). The code used to generate the results in this study is available at https://github.com/albertozenere/GraMS. Any additional information is available from the lead contact upon request.

## Abbreviations

BP: Before pregnancy
CNS: Central nervous system
DEGs: Differentially expressed genes
DMPs: Differentially methylated probes
DPBS: Dulbecco’s phosphate-buffered saline
FBS: Fetal bovine serum
FDR: False discovery rate
gw: Gestational week
HC: Healthy controls
IMDM: Iscove’s modified Dulbecco’s medium
IVF: In vitro fertilization
MS: Multiple sclerosis
OR: Odds ratio
P4: Progesterone
PBMCs: Peripheral blood mononuclear cells
PP: Post-partum
PPI: Protein–protein interaction
RA: Rheumatoid arthritis
RIN: RNA integrity
RNA-seq: RNA sequencing
RT: Room temperature

## References

[1] Dendrou CA, Fugger L, Friese MA (2015). Immunopathology of multiple sclerosis. Nat Rev Immunol.

[2] Confavreux C, Hutchinson M, Hours MM, Cortinovis-Tourniaire P, Moreau T (1998). Rate of pregnancy-related relapse in multiple sclerosis. N Engl J Med.

[3] Finkelsztejn A, Brooks J, Paschoal F, Fragoso Y (2011). What can we really tell women with multiple sclerosis regarding pregnancy? A systematic review and meta-analysis of the literature. BJOG Int J Obstet Gynaecol.

[4] Mor G, Aldo P, Alvero AB (2017). The unique immunological and microbial aspects of pregnancy. Nat Rev Immunol.

[5] Aghaeepour N, Ganio EA, Mcilwain D, Tsai AS, Tingle M, Van Gassen S (2017). An immune clock of human pregnancy. Sci Immunol..

[6] Ysrraelit MC, Correale J (2019). Impact of sex hormones on immune function and multiple sclerosis development. Immunology.

[7] Shah NM, Lai PF, Imami N, Johnson MR (2019). Progesterone-related immune modulation of pregnancy and labor. Front Endocrinol.

[8] Kieffer TEC, Laskewitz A, Scherjon SA, Faas MM, Prins JR (2019). Memory T cells in pregnancy. Front Immunol.

[9] Lissauer D, Kilby MD, Moss P (2017). Maternal effector T cells within decidua: the adaptive immune response to pregnancy?. Placenta.

[10] Attfield KE, Jensen LT, Kaufmann M, Friese MA, Fugger L. The immunology of multiple sclerosis. Nat Rev Immunol. 2022;22:734–50. 10.1038/s41577-022-00718-z 35508809

[11] Saligrama N, Zhao F, Sikora MJ, Serratelli WS, Fernandes RA, Louis DM (2019). Opposing T cell responses in experimental autoimmune encephalomyelitis. Nature.

[12] Badam TV, Hellberg S, Mehta RB, Lechner-Scott J, Lea RA, Tost J, et al. CD4^+^ T-cell DNA methylation changes during pregnancy significantly correlate with disease-associated methylation changes in autoimmune diseases. Epigenetics. 2022;17:1040–55. 10.1080/15592294.2021.1982510 PMC9487751 34605719

[13] Ramien C, Yusko EC, Engler JB, Gamradt S, Patas K, Schweingruber N (2019). T cell repertoire dynamics during pregnancy in multiple sclerosis. Cell Rep.

[14] Spadaro M, Martire S, Marozio L, Mastromauro D, Montanari E, Perga S (2019). Immunomodulatory effect of pregnancy on leukocyte populations in patients with multiple sclerosis: a comparison of peripheral blood and decidual placental tissue. Front Immunol.

[15] Koetzier SC, Neuteboom RF, Wierenga-Wolf AF, Melief M-J, de Mol CL, van Rijswijk A (2021). Effector T helper cells are selectively controlled during pregnancy and related to a postpartum relapse in multiple sclerosis. Front Immunol.

[16] Engler JB, Heckmann NF, Jäger J, Gold SM, Friese MA (2019). Pregnancy enables expansion of disease-specific regulatory T cells in an animal model of multiple sclerosis. J Immunol.

[17] Langer-Gould A, Gupta R, Huang S, Hagan A, Atkuri K, Leimpeter AD (2010). Interferon-gamma-producing T cells, pregnancy, and postpartum relapses of multiple sclerosis. Arch Neurol.

[18] Neuteboom RF, Verbraak E, Wierenga-Wolf AF, van Meurs M, Steegers EA, de Groot CJ (2010). Pregnancy-induced fluctuations in functional T-cell subsets in multiple sclerosis patients. Mult Scler J.

[19] Gilli F, Lindberg RLP, Valentino P, Marnetto F, Malucchi S, Sala A (2010). Learning from nature: pregnancy changes the expression of inflammation-related genes in patients with multiple sclerosis. PLoS ONE.

[20] Airas L, Nikula T, Huang Y-H, Lahesmaa R, Wiendl H (2007). Postpartum-activation of multiple sclerosis is associated with down-regulation of tolerogenic HLA-G. J Neuroimmunol.

[21] Iannello A, Rolla S, Maglione A, Ferrero G, Bardina V, Inaudi I (2019). Pregnancy epigenetic signature in T Helper 17 and T regulatory cells in multiple sclerosis. Front Immunol.

[22] Piñero J, Saüch J, Sanz F, Furlong LI (2021). The DisGeNET cytoscape app: exploring and visualizing disease genomics data. Comput Struct Biotechnol J.

[23] International multiple sclerosis genetics consortium (2019). Multiple sclerosis genomic map implicates peripheral immune cells and microglia in susceptibility. Science.

[24] Ghiassian SD, Menche J, Barabási A-L (2015). A DIseAse MOdule Detection (DIAMOnD) algorithm derived from a systematic analysis of connectivity patterns of disease proteins in the human interactome. PLOS Comput Biol.

[25] de Weerd HA, Badam TVS, Martínez-Enguita D, Åkesson J, Muthas D, Gustafsson M (2020). MODifieR: an Ensemble R Package for inference of disease modules from transcriptomics networks. Bioinformatics.

[26] Hellberg S, Raffetseder J, Rundquist O, Magnusson R, Papapavlou G, Jenmalm MC (2021). Progesterone dampens immune responses in in vitro activated CD4+ T cells and affects genes associated with autoimmune diseases that improve during pregnancy. Front Immunol.

[27] Ghaemi MS, DiGiulio DB, Contrepois K, Callahan B, Ngo TTM, Lee-McMullen B (2019). Multiomics modeling of the immunome, transcriptome, microbiome, proteome and metabolome adaptations during human pregnancy. Bioinformatics.

[28] Papapavlou Lingehed G, Hellberg S, Huang J, Khademi M, Kockum I, Carlsson H (2022). Plasma protein profiling reveals dynamic immunomodulatory changes in multiple sclerosis patients during pregnancy. Front Immunol.

[29] Kourtis AP, Read JS, Jamieson DJ (2014). Pregnancy and Infection. N Engl J Med.

[30] Mittal A, Pachter L, Nelson JL, Kjærgaard H, Smed MK, Gildengorin VL (2015). Pregnancy-induced changes in systemic gene expression among healthy women and women with rheumatoid arthritis. PLoS ONE.

[31] Erlebacher A (2013). Mechanisms of T cell tolerance towards the allogeneic fetus. Nat Rev Immunol.

[32] Abu-Raya B, Michalski C, Sadarangani M, Lavoie PM (2020). Maternal immunological adaptation during normal pregnancy. Front Immunol.

[33] Chen D, Wang W, Wu L, Liang L, Wang S, Cheng Y (2022). Single-cell atlas of peripheral blood mononuclear cells from pregnant women. Clin Transl Med.

[34] Hellberg S, Eklund D, Gawel DR, Köpsén M, Zhang H, Nestor CE (2016). Dynamic response genes in CD4+ T cells reveal a network of interactive proteins that classifies disease activity in multiple sclerosis. Cell Rep.

[35] Cappelletti C, Eriksson A, Brorson IS, Leikfoss IS, Kråbøl O, Høgestøl EA (2022). Quantitative proteomics reveals protein dysregulation during T cell activation in multiple sclerosis patients compared to healthy controls. Clin Proteomics.

[36] Rundquist O, Nestor CE, Jenmalm MC, Hellberg S, Gustafsson M (2022). Progesterone inhibits the establishment of activation-associated chromatin during TH1 differentiation. Front Immunol.

[37] Papapavlou G, Hellberg S, Raffetseder J, Brynhildsen J, Gustafsson M, Jenmalm MC (2021). Differential effects of estradiol and progesterone on human T cell activation in vitro. Eur J Immunol.

[38] Hughes GC, Clark EA, Wong AH (2013). The intracellular progesterone receptor regulates CD4+ T cells and T cell-dependent antibody responses. J Leukoc Biol.

[39] Chien EJ, Chang C-P, Lee W-F, Su T-H, Wu C-H (2006). Non-genomic immunosuppressive actions of progesterone inhibits PHA-induced alkalinization and activation in T cells. J Cell Biochem.

[40] Mohammad I, Starskaia I, Nagy T, Guo J, Yatkin E, Väänänen K (2018). Estrogen receptor α contributes to T cell-mediated autoimmune inflammation by promoting T cell activation and proliferation. Sci Signal..

[41] Goodman WA, Bedoyan SM, Havran HL, Richardson B, Cameron MJ, Pizarro TT (2020). Impaired estrogen signaling underlies regulatory T cell loss-of-function in the chronically inflamed intestine. Proc Natl Acad Sci.

[42] Langer-Gould A (2016). Sex hormones and multiple sclerosis: another informative failure. Lancet Neurol.

[43] Dobin A, Davis CA, Schlesinger F, Drenkow J, Zaleski C, Jha S (2013). STAR: ultrafast universal RNA-seq aligner. Bioinformatics.

[44] Patro R, Duggal G, Love MI, Irizarry RA, Kingsford C (2017). Salmon provides fast and bias-aware quantification of transcript expression. Nat Methods.

[45] Tian Y, Morris TJ, Webster AP, Yang Z, Beck S, Feber A (2017). ChAMP: updated methylation analysis pipeline for Illumina BeadChips. Bioinformatics.

[46] Zhou W, Laird PW, Shen H (2017). Comprehensive characterization, annotation and innovative use of Infinium DNA methylation BeadChip probes. Nucleic Acids Res.

[47] Teschendorff AE, Marabita F, Lechner M, Bartlett T, Tegner J, Gomez-Cabrero D (2013). A beta-mixture quantile normalization method for correcting probe design bias in Illumina Infinium 450 k DNA methylation data. Bioinforma Oxf Engl.

[48] Johnson WE, Li C, Rabinovic A (2007). Adjusting batch effects in microarray expression data using empirical Bayes methods. Biostat Oxf Engl.

[49] Zhang Y, Parmigiani G, Johnson WE (2020). ComBat-seq: batch effect adjustment for RNA-seq count data. NAR Genomics Bioinforma..

[50] Robinson MD, McCarthy DJ, Smyth GK (2010). edgeR: a Bioconductor package for differential expression analysis of digital gene expression data. Bioinformatics.

[51] Ritchie ME, Phipson B, Wu D, Hu Y, Law CW, Shi W (2015). limma powers differential expression analyses for RNA-sequencing and microarray studies. Nucleic Acids Res.

[52] Du P, Zhang X, Huang C-C, Jafari N, Kibbe WA, Hou L (2010). Comparison of Beta-value and M-value methods for quantifying methylation levels by microarray analysis. BMC Bioinformatics.

[53] Komori HK, Hart T, LaMere SA, Chew PV, Salomon DR (1950). Defining CD4 T cell memory by the epigenetic landscape of CpG DNA methylation. J Immunol Baltim Md.

[54] Durek P, Nordström K, Gasparoni G, Salhab A, Kressler C, de Almeida M (2016). Epigenomic profiling of human CD4+ T cells supports a linear differentiation model and highlights molecular regulators of memory development. Immunity.

[55] Law CW, Chen Y, Shi W, Smyth GK (2014). voom: precision weights unlock linear model analysis tools for RNA-seq read counts. Genome Biol.

[56] Szklarczyk D, Gable AL, Nastou KC, Lyon D, Kirsch R, Pyysalo S (2021). The STRING database in 2021: customizable protein-protein networks, and functional characterization of user-uploaded gene/measurement sets. Nucleic Acids Res.

[57] Shannon P, Markiel A, Ozier O, Baliga NS, Wang JT, Ramage D (2003). Cytoscape: a software environment for integrated models of biomolecular interaction networks. Genome Res.

[58] Yu G, Wang L-G, Han Y, He Q-Y (2012). clusterProfiler: an R Package for comparing biological themes among gene clusters. OMICS J Integr Biol..

[59] Yu G, Wang L-G, He Q-Y (2015). ChIPseeker: an R/Bioconductor package for ChIP peak annotation, comparison and visualization. Bioinformatics.

