# Supplementary material for: Prominent epigenetic and transcriptomic changes in CD4+ and CD8+ T cells during and after pregnancy in women with multiple sclerosis and controls
Source: J Neuroinflammation. 2023 Apr 27;20:98. doi: 10.1186/s12974-023-02781-2 (PMC10134602; doi:10.1186/s12974-023-02781-2)
Supplement: Supplementary file 1 — Additional file 1: Figure S1. Overview of the experimental set up. Figure S2. Overview of the analysis workflow from initial raw data to differential analysis and module inference. Rebound DEGs/DMPs were defined as the overlap between the differentially expressed genes/differentially methylated probes identified in the 3rd trimesterand post-partumsimultaneously, calculated for MS and HC separately. Genes associated with at least one rebound DMP were denoted rebound DMGs. Rebound DEGs/DMGs in common between MS and HC were termed shared rebound DEGs/DMGs. These genes served as input for creating RNA-seq and methylation modules for each cell type separately. The RNA-seq and methylation modules were overlapped to create one CD4+ rebound pregnancy module and one CD8+ rebound pregnancy module. DEGs and DMPs with a nominal p-value ≤0.05were included for analysis. DEG, differentially expressed gene; DMG, differentially methylated gene; DMP, differentially methylated probe; MMD, mean methylation difference. Figure S3. Overview of the samples used for RNA sequencing and DNA methylation from women with MS and healthy controls. A number of samples were excluded for the RNA-sequencing due to technical issues at the sequencing facility. Two samples were also excluded after sequencing due to insufficient sequencing depth. The number of samples is stated as the number of resting/activatedor resting cells alonein the two lower panels. HC, healthy controls; MS, Multiple sclerosis. Figure S4. DNA and RNA extracted from CD4+ and CD8+ T cells from women with MS and healthy controlswere analyzed by RNA-seq and Infinium Methylation EPIC 850 K for DNA methylation. Shown are the correlation between gene countsand the beta values of all detected CpGsfor the comparisons 3rd-2nd trimester and 2nd -1st trimester for resting CD4+ cells and CD8+ cells in women with MS and HC. Pearson’s correlation r is shown in the individual graphs for each comparison. All correlations had a p < 2.2 x 10^-16^. HC, [file 12974_2023_2781_MOESM1_ESM.docx]

**
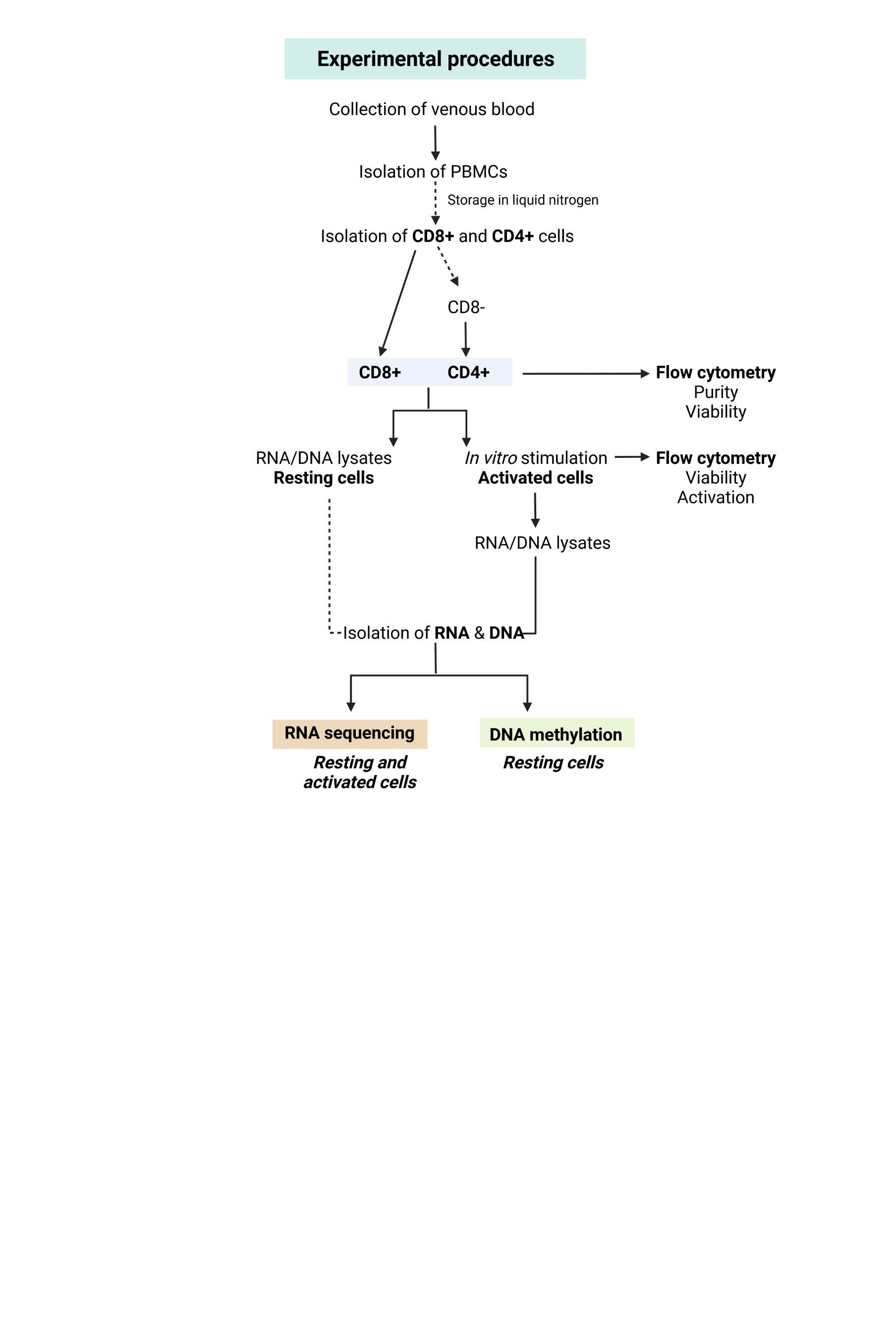
**

**Figure S1.** Overview of the experimental set up.

**
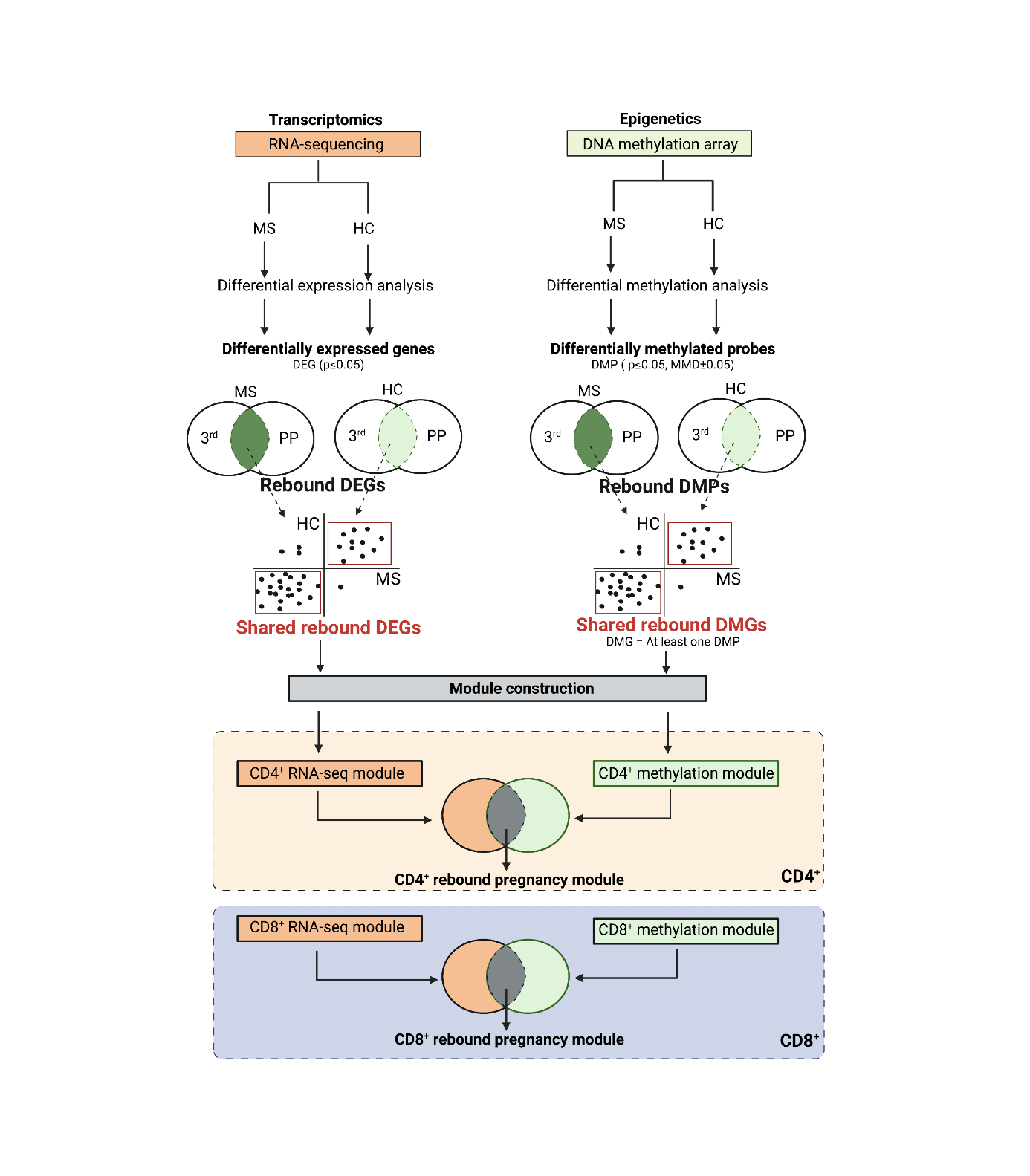
Figure S2.** **Overview of the analysis workflow from initial raw data to differential analysis and module inference.** Rebound DEGs/DMPs were defined as the overlap between the differentially expressed genes/differentially methylated probes identified in the 3^rd^ trimester (3^rd^-1^st^) and postpartum (PP-3^rd^) simultaneously, calculated for MS and HC separately. Genes associated with at least one rebound DMP were denoted rebound DMGs. Rebound DEGs/DMGs in common between MS and HC were termed shared rebound DEGs/DMGs. These genes served as input for creating RNA-seq and methylation modules for each cell type separately. The RNA-seq and methylation modules were overlapped to create one CD4^+^ rebound pregnancy module and one CD8^+^ rebound pregnancy module. DEGs and DMPs with a nominal p-value ≤0.05 (and an MMD±0.05 for DMPs) were included for analysis. DEG, differentially expressed gene; DMG, differentially methylated gene; DMP, differentially methylated probe; MMD, mean methylation difference.

**
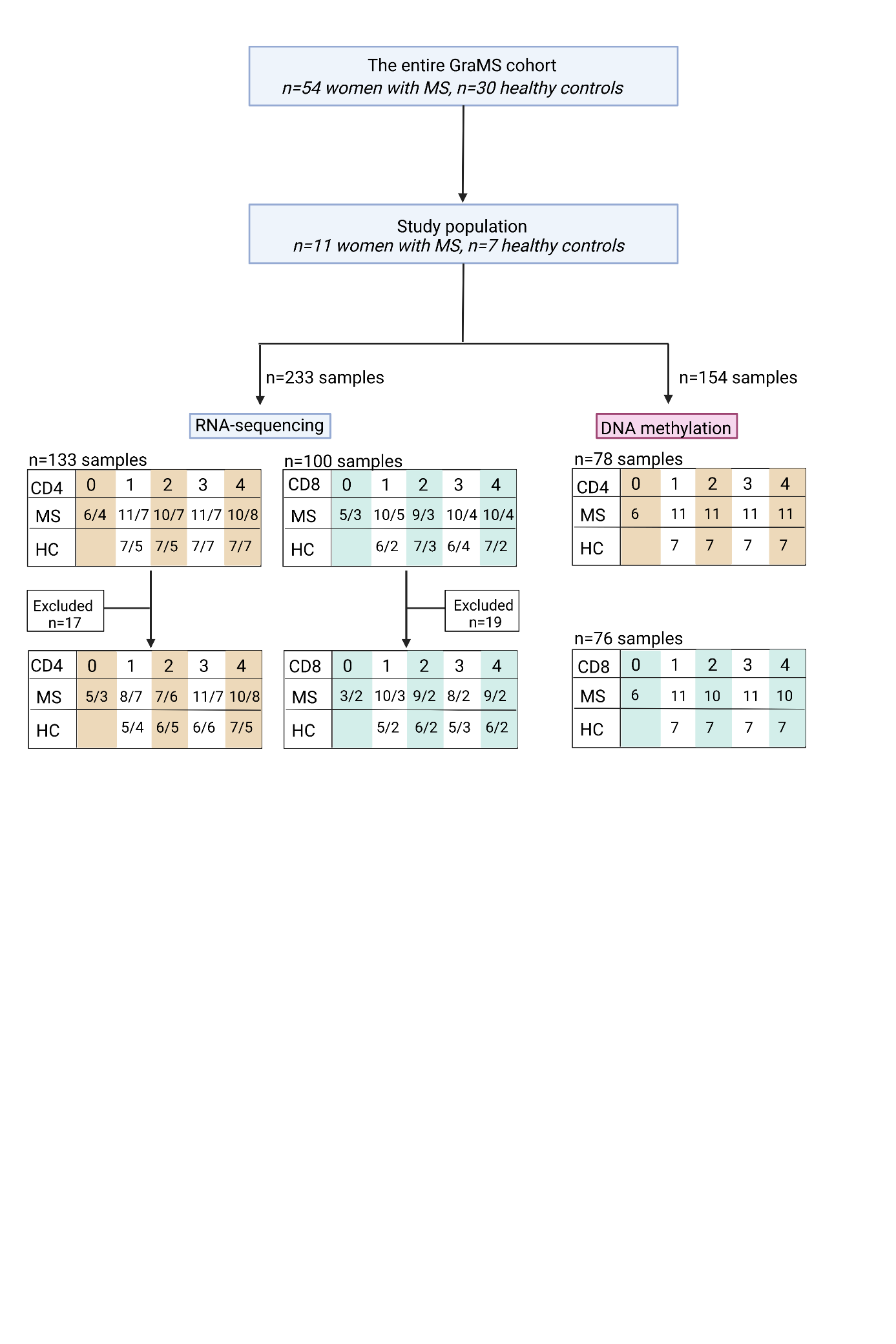
**

**Figure S3.** Overview of the samples used for RNA sequencing and DNA methylation from women with MS *~~patients~~* and healthy controls (HC). A number of samples were excluded for the RNA-sequencing due to technical issues at the sequencing facility. Two samples were also excluded after sequencing due to insufficient sequencing depth. The number of samples is stated as the number of resting/activated (RNA-sequencing) or resting cells alone (DNA methylation) in the two lower panels. HC, healthy controls; MS, Multiple sclerosis.

**
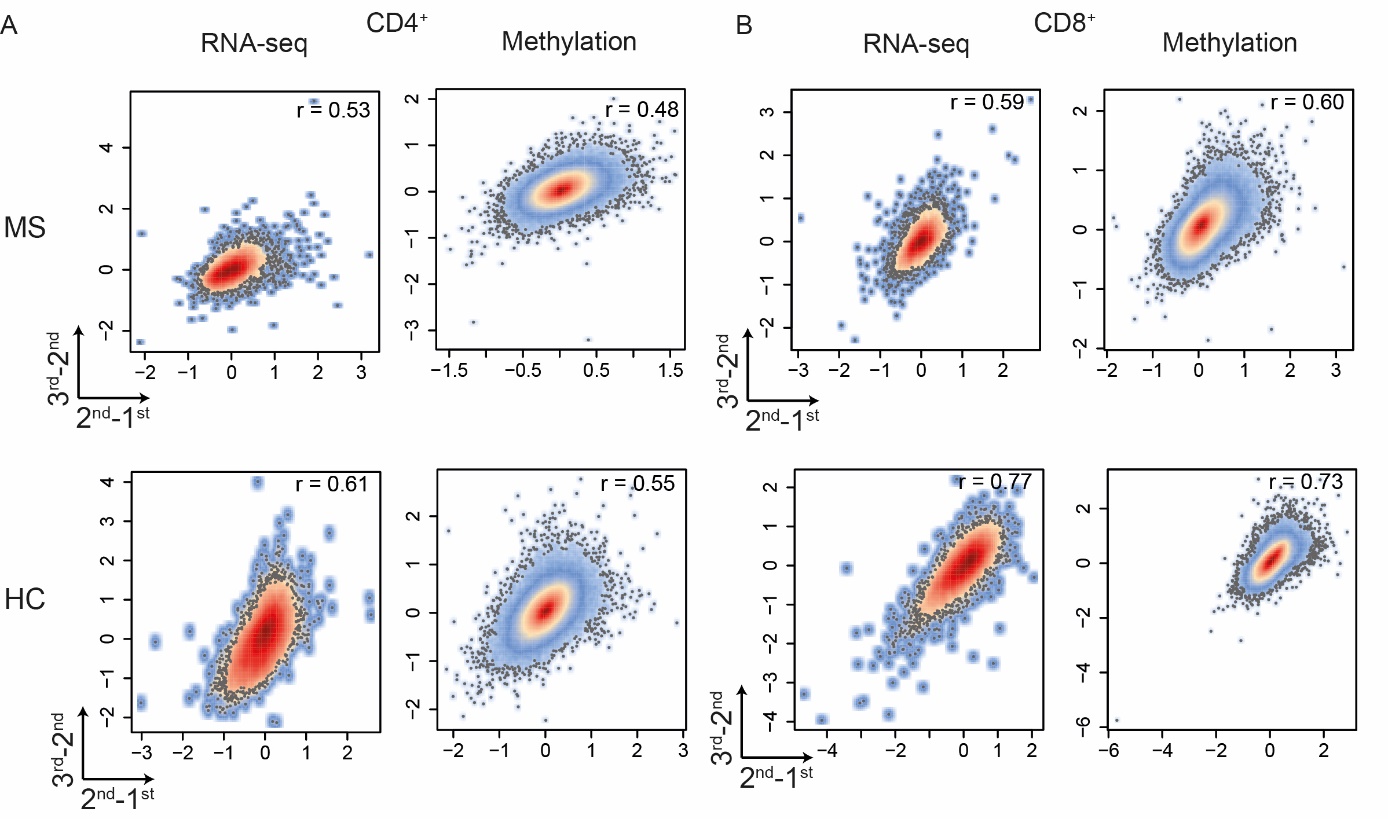
Figure S4.** DNA and RNA extracted from CD4^+^ and CD8^+^ T cells from women with MS and healthy controls (HC) were analyzed by RNA-seq and Infinium MethylationEPIC 850K for DNA methylation. Shown are the correlation between gene counts (for RNA-seq) and the beta values of all detected CpGs (for methylation) for the comparisons 3^rd^-2^nd^ trimester and 2^nd^ -1^st^ trimester for resting **(A)** CD4^+^ cells and **(B)** CD8^+^ cells in women with MS and HC. Pearson’s correlation r is shown in the individual graphs for each comparison. All correlations had a p<2.2x10^-16^. HC, healthy controls; MS, multiple sclerosis.

**
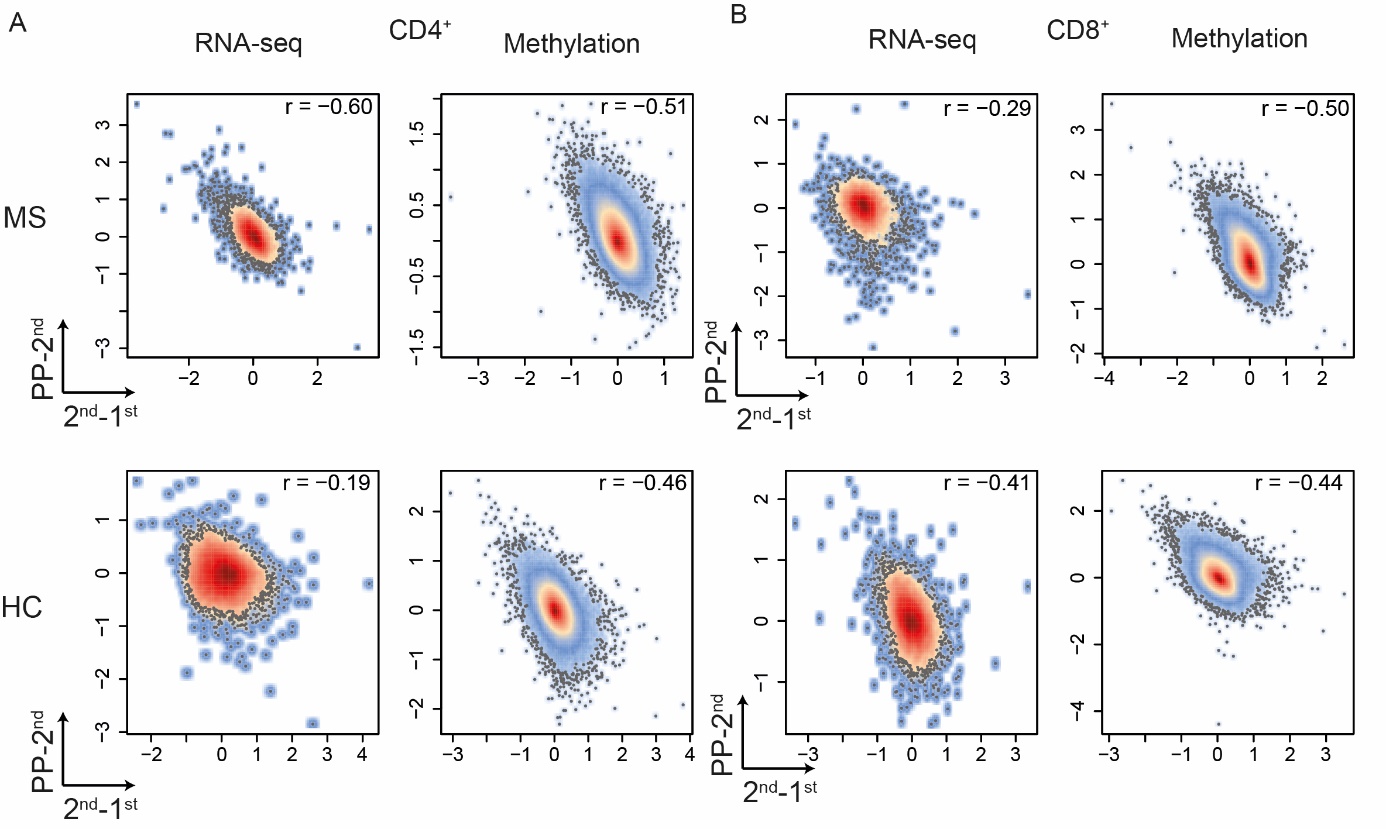
Figure S5.** DNA and RNA extracted from CD4^+^ and CD8^+^ T cells from women with MS and healthy controls (HC) were analyzed by RNA-seq and Infinium MethylationEPIC 850K for DNA methylation. The correlation between gene counts (for RNA-seq) and the beta values of all detected CpGs (for methylation) between PP-2^nd^ trimester and 2^nd^-1^st^ trimester for resting **(A)** CD4^+^ cells and **(B)** CD8^+^ cells in women with MS and HC. Pearson’s correlation r is shown in the individual graphs for each comparison. All correlations had a p<2.2x10^-16^. HC, healthy controls; MS, multiple sclerosis, PP; post-partum.

**
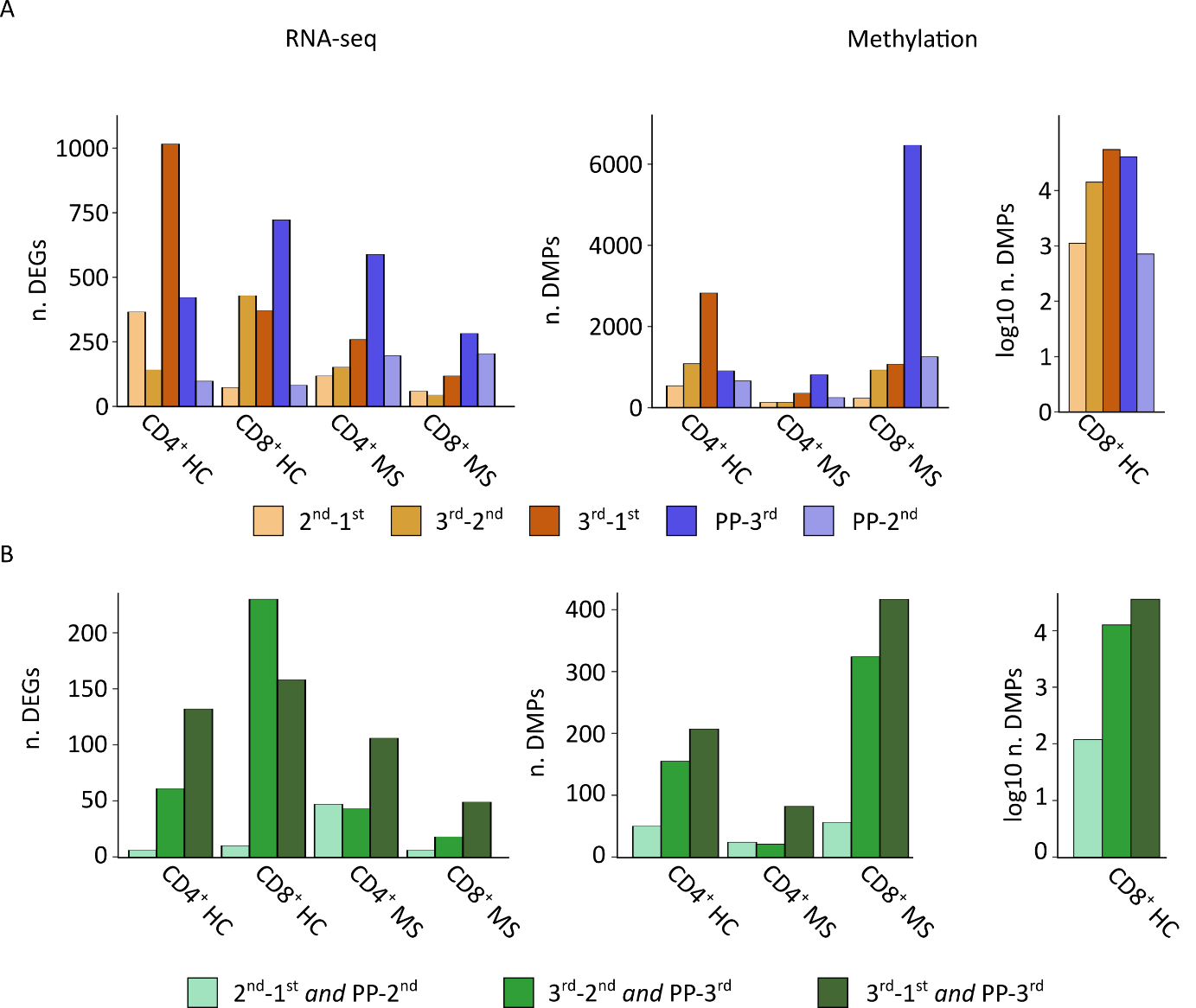
Figure S6. (A)** Number of nominally differentially expressed genes (DEGs) and differentially methylated CpGs (DMPs) during pregnancy and post-partum comparing 2^nd^-1^st^, 3^rd^-2^nd^ , 3^rd^-1^st^, PP-3^rd^ and PP-2^nd^ (p≤0.05). **(B)** Number of overlapping genes and CpGs during pregnancy and after pregnancy*, i.e.,* PP-2^nd^ compared to 2^nd^-1^st^ and PP-3^rd^ compared to 3^rd^-2^nd^ and 3^rd^-1^st^ trimesters. DEGs; differentially expressed genes, DMPs; differentially methylated probes, HC; healthy controls, MS; multiple sclerosis, PP; post-partum.

**
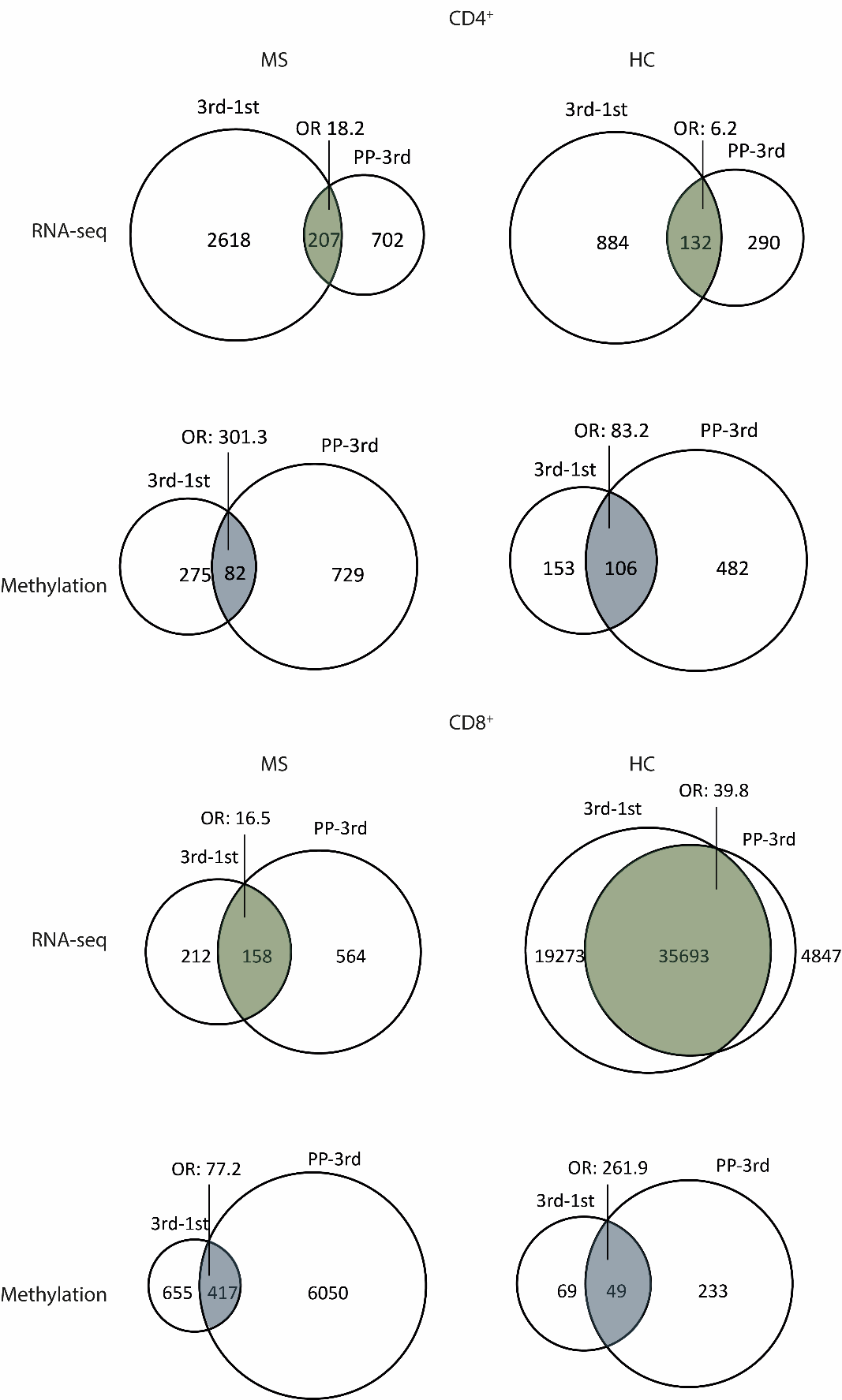
**

**Figure S7.** Venn diagrams showing the overlap between the nominally differentially expressed genes or differentially methylated CpGs (p≤0.05) during (3^rd^-1^st^) and after pregnancy (PP-3^rd^) in women with MS and healthy controls (HC). Fisher’s Exact Test was used to calculate the enrichment of the overlaps. Odds ratios are shown, and all overlaps had a p<10^-16^. HC; healthy controls, MS; multiple sclerosis, OR; odds ratio, PP; post-partum.


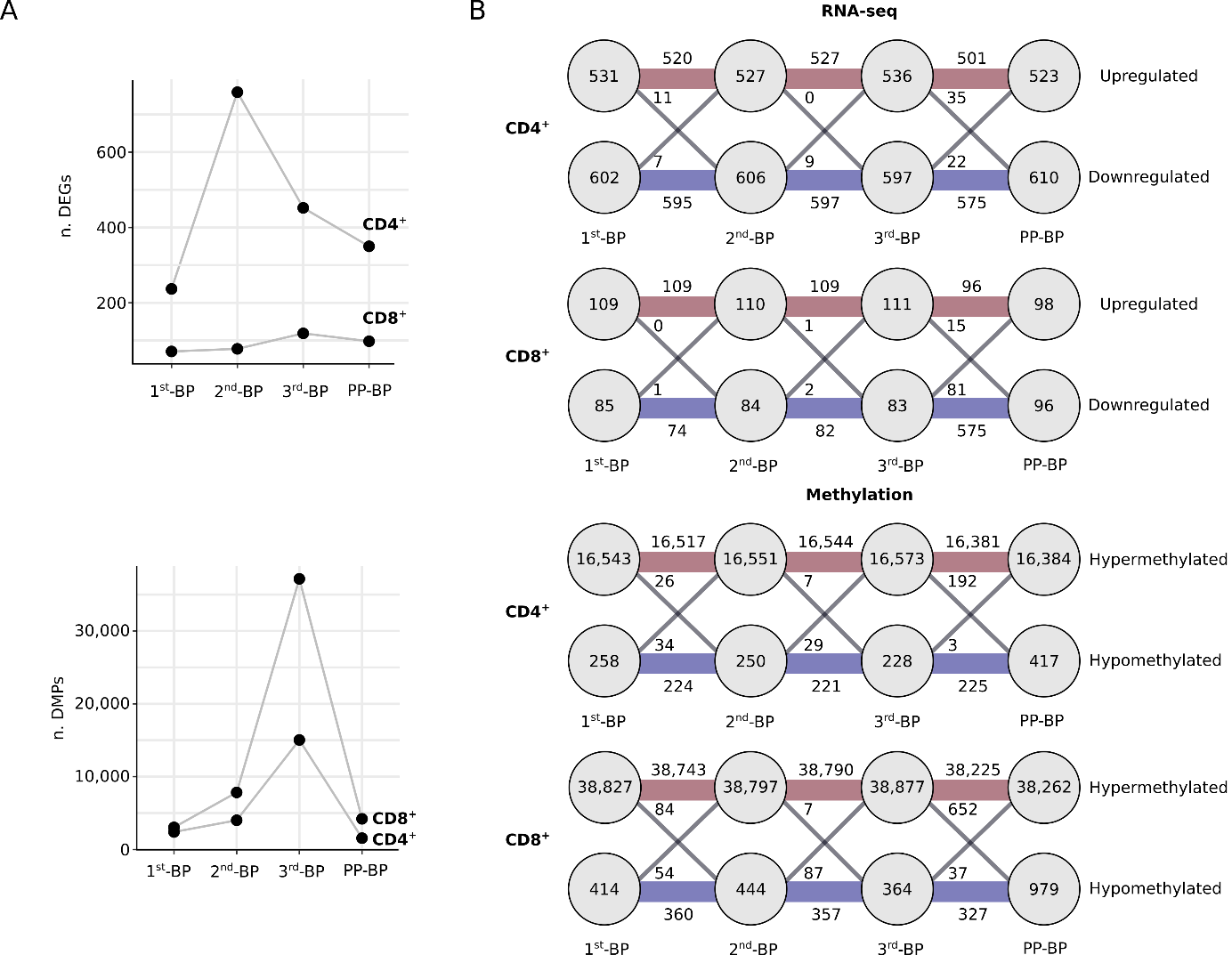


**Figure S8.** Differential analysis between the samples collected before pregnancy and the remaining time points. (A) Number of DMPs and DEGs (p≤0.05) for each comparison. (B) Number of probes and genes that are hypermethylated/hypomethylated (respectively upregulated/downregulated) in each comparison. BP, before pregnancy; PP, post-partum; DEG, differentially expressed gene; DMP, differentially methylated probe.


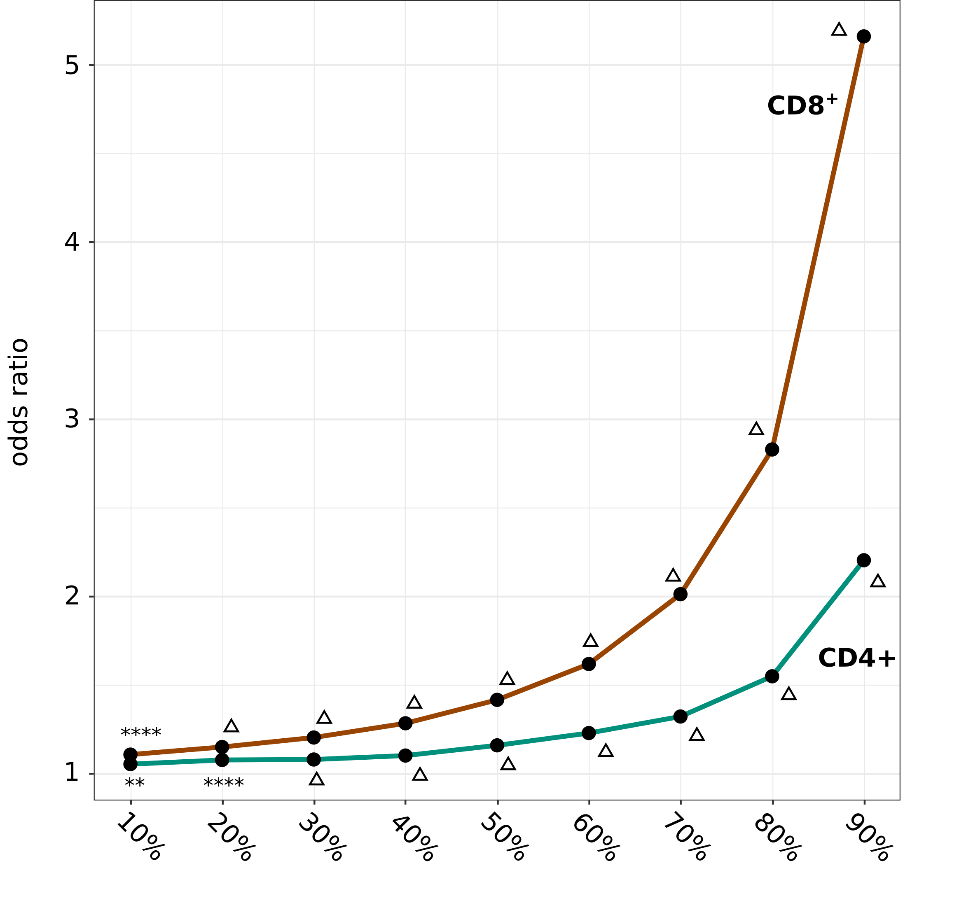


**Figure S9.** Regulatory patterns between DNA methylation and gene expression are conserved between MS and HC. The regulation exerted by each CpG was measured by the Spearman correlation coefficient with the respective gene, as annotated by Illumina. Correlations were computed independently in CD4^+^ and CD8^+^, for MS and HC. The correlations obtained in each of the four groups were divided into deciles based on their absolute values. The overlap between CpG-gene pairs that belong to the same decile in both MS and HC was carried out using Fisher’s exact test. **p<0.01, ****p<0.0001, Δ p<10^-16^.


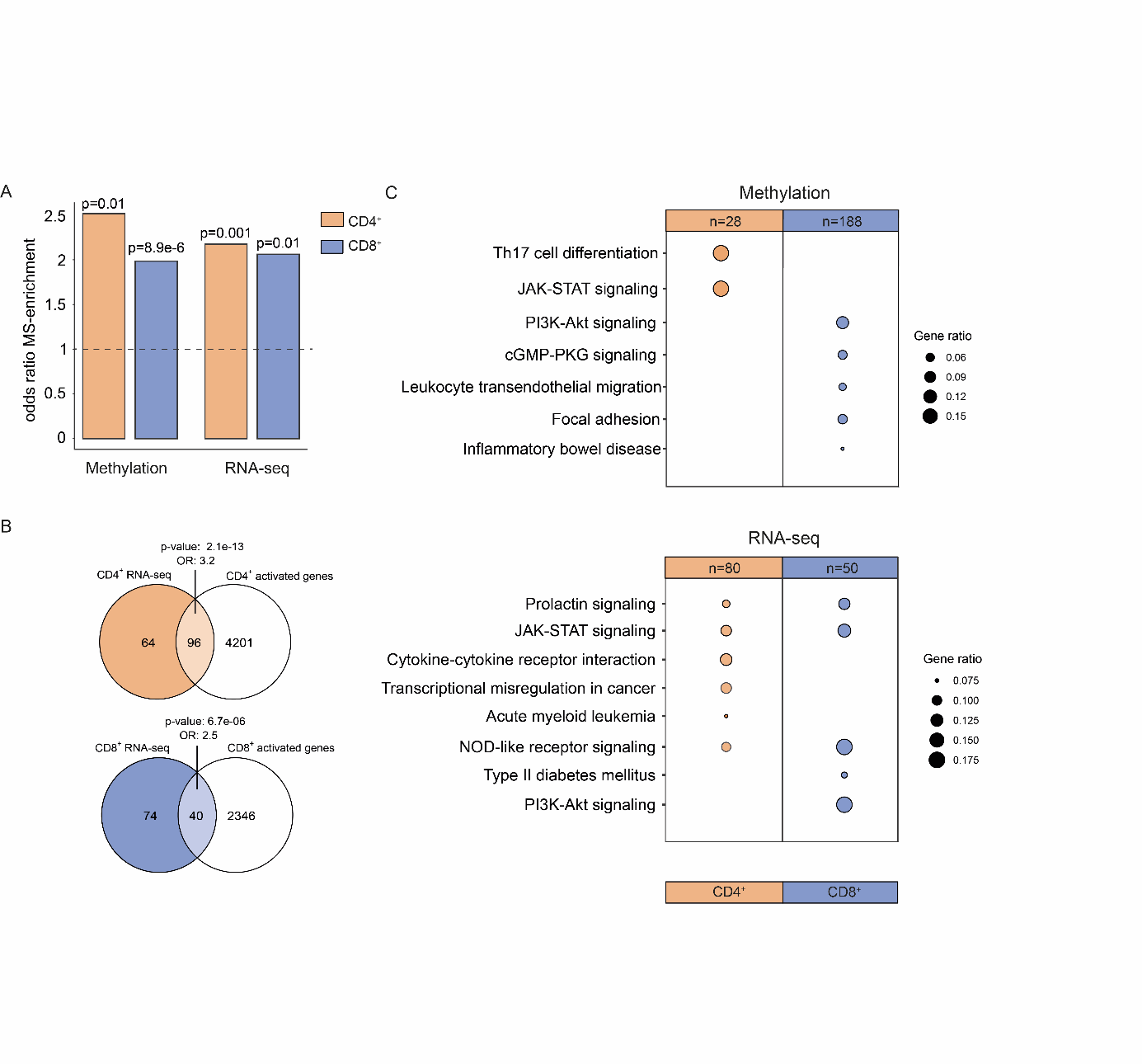


**Figure S10.** The CD4^+^ and CD8^+^ rebound genes were derived by overlapping (1) the differentially expressed genes (DEGs: from RNA-seq) from 3^rd^-1^st^ trimester and PP-3^rd^ in both women with MS *~~patients~~* and healthy controls (HC) and (2) Genes (derived from the DMPs for the methylation analysis) for the same comparisons. These genes were later used to infer modules. **(A)** Enrichment of MS-associated genes based on GWAS-derived MS genes and DisGeNET; p denotes p-value. **(B)** Overlap between the genes significantly affected by activation, performed only on the genes derived from the RNA-seq analysis. **(C)** KEGG pathway enrichment. Shown are the top 5 pathways (in terms of adjusted p-value) of both groups. Number of genes (n) is shown above. Enrichment was calculated using Fisher’s exact test and p≤0.05 was considered statistically significant. DEGs; differentially expressed genes, DMPs; differentially methylated probes, MS; multiple sclerosis. Shown are the top 5 pathways (in terms of adjusted p-value) of both groups, with adjusted p-value≤0.05.


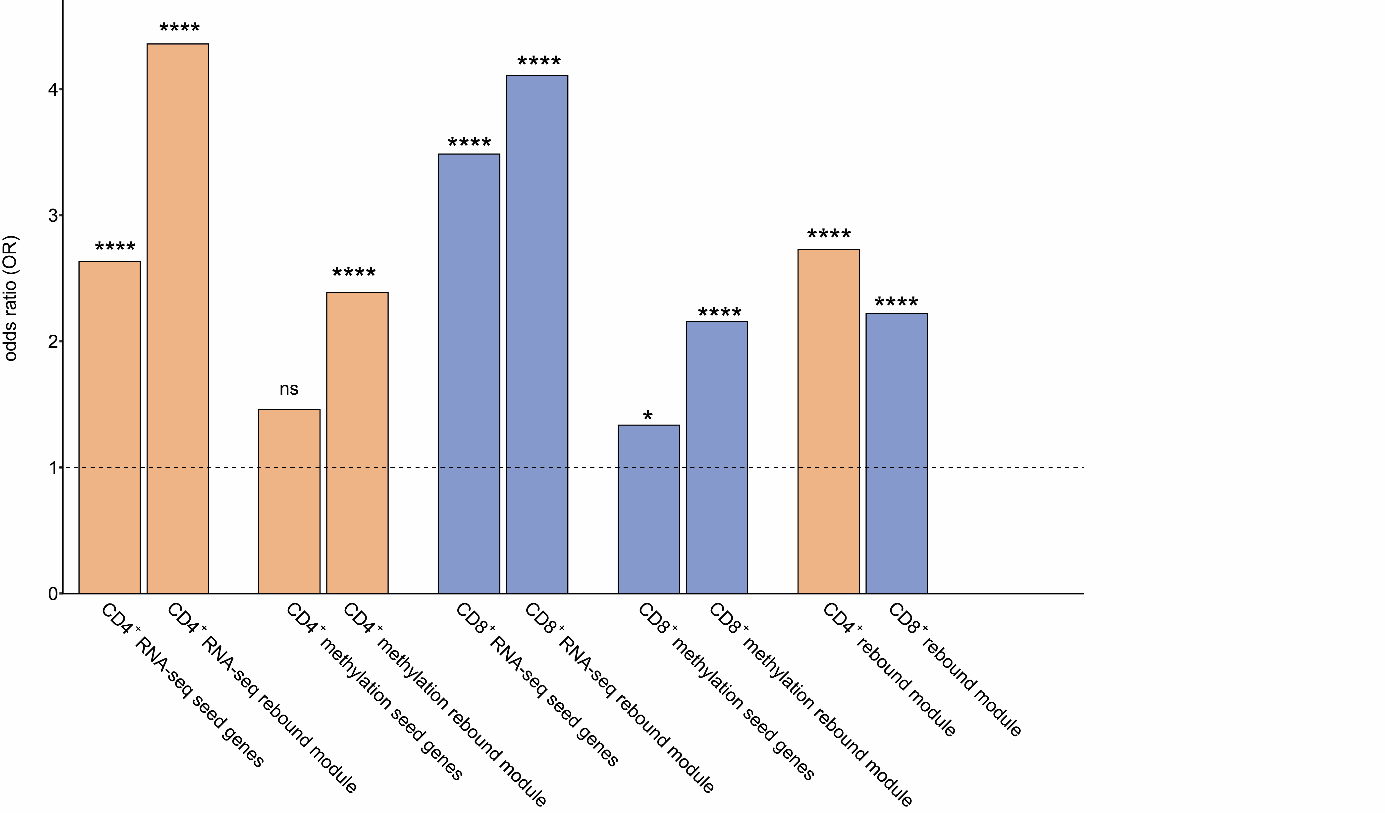


**Figure S11.** Enrichment of P4-associated genes. The rebound seed genes (used as input for module inference), the resulting module genes for each omic and the shared genes derived from combining the modules for both RNA-seq and methylation for each cell type (CD4^+^/CD8^+^ rebound pregnancy modules) were tested for enrichment of P4-associated genes using Fisher’s exact test. The P4-associated genes (n=1,992) were derived from Hellberg *et al.,* Front Immunol (2021). ns; non-significant, P4; progesterone. *p<0.05, ****p<0.0001.


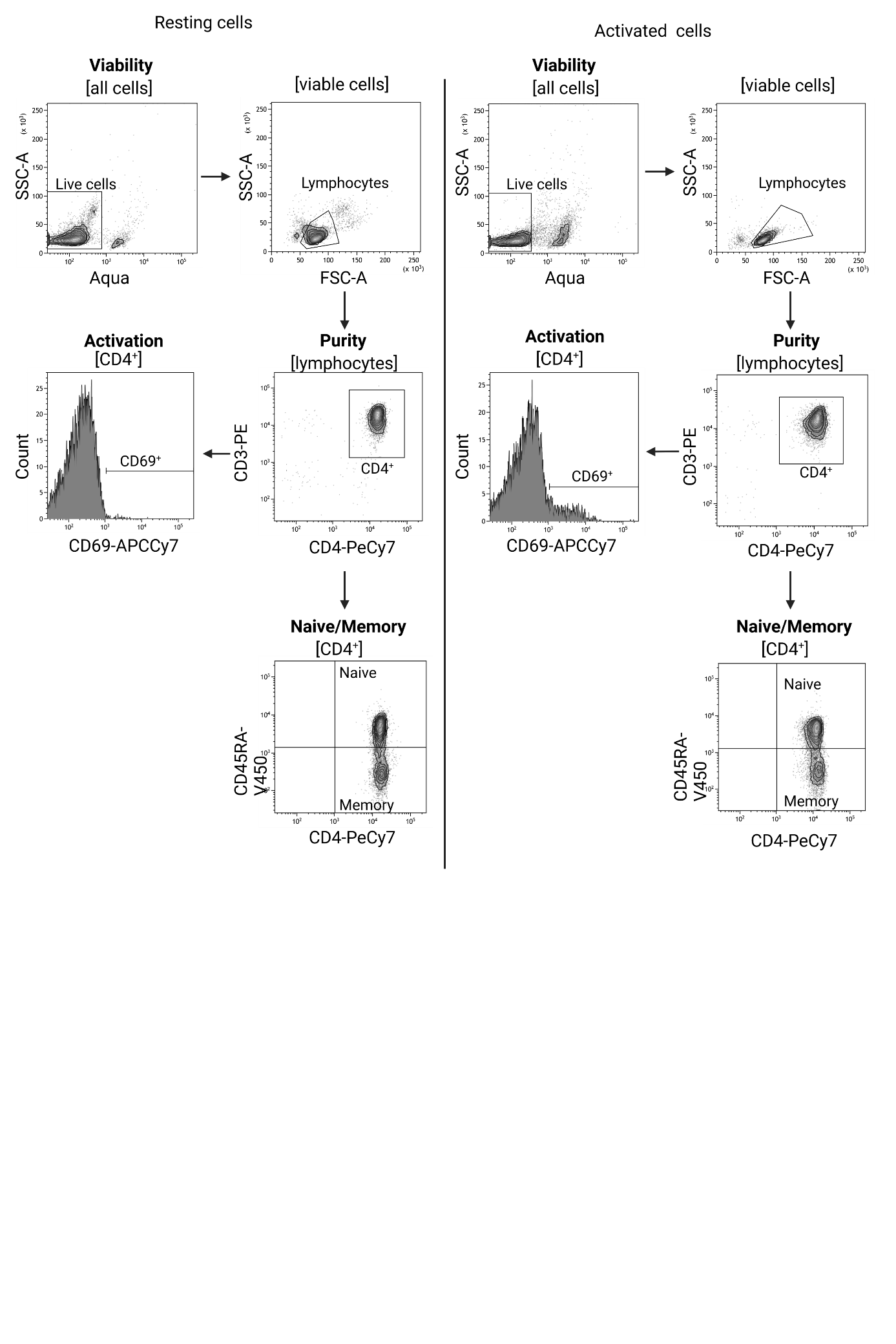


**Figure S12.** Flow cytometry characterization of resting and activated CD4^+^ T cells. Flow cytometry gating strategies to assess purity, viability, and activation status in resting and activated CD4^+^ T cells. Viable cells were identified as Aqua^-^ and further gated based on forward (FSC) and side (SSC) scatter to further characterize CD4^+^ cells. The cut-off value for CD69 expression was based on the expression in the resting cells. Definition of naïve and memory T cells was based on the contour of the CD45RA^+^ (naïve) and CD45RA^-^ (memory) populations. Viability, purity of the isolated cells, activation level (based on CD69 expression) and proportion of naïve and memory CD4+ T cells were evaluated on resting cells on day 0. Activated cells were analyzed for viability, CD69 expression (activation) and proportion of naïve and memory cells. The figure shows one representative sample.


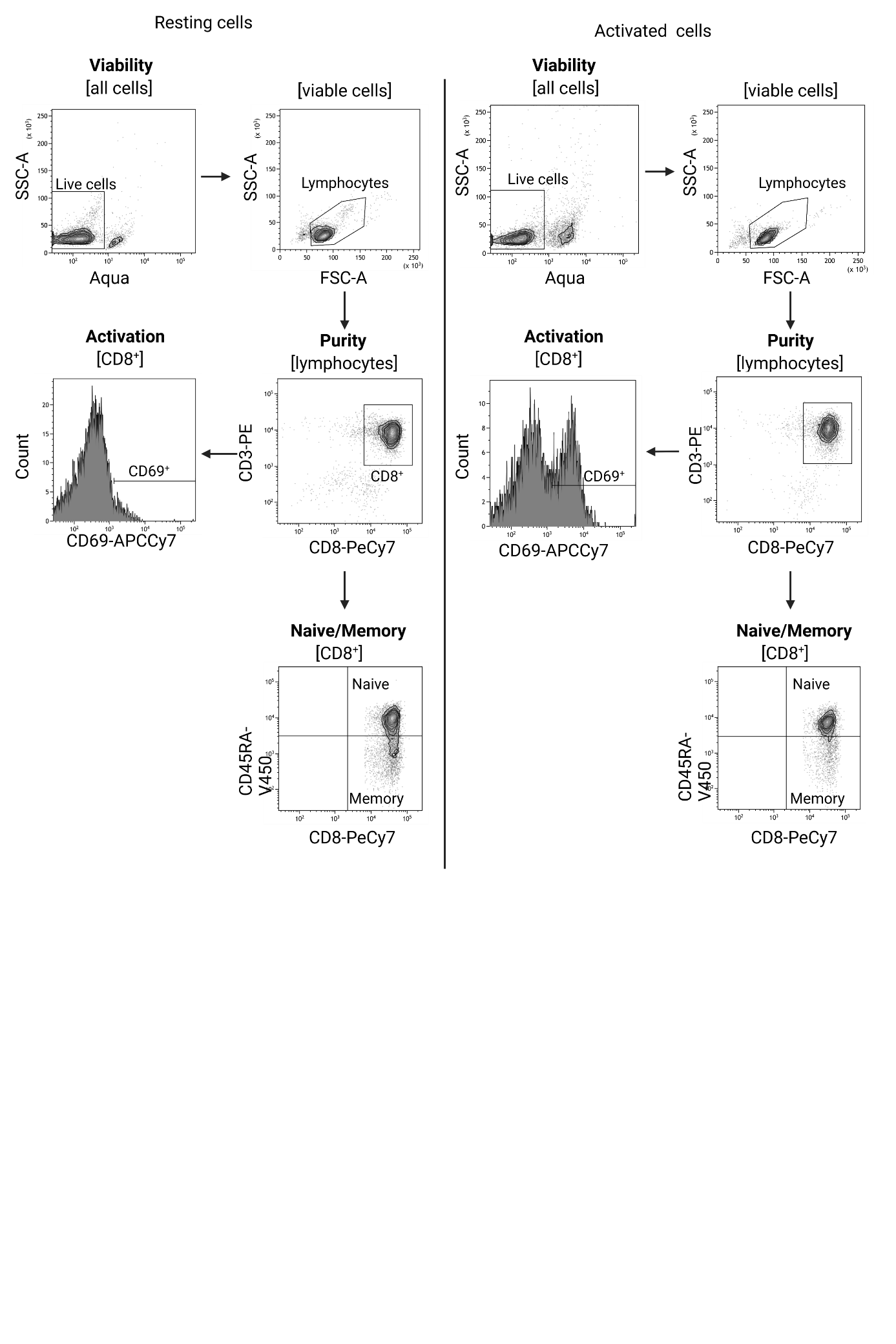


**Figure S13.** Flow cytometry characterization of resting and activated CD8^+^ T cells. Flow cytometry gating strategies to assess purity, viability, and activation status in resting and activated CD8^+^ T cells. Viable cells were identified as Aqua^-^ and further gated based on forward (FSC) and side (SSC) scatter and to characterize CD8^+^ cells. The cut-off value for CD69 expression was based on the expression in the resting cells. Definition of naïve and memory was based on the contour of the CD45RA^+^ (naïve) and CD45RA^-^ populations. Viability, purity of the isolated cells, activation level (based on CD69 expression) and proportion of naïve and memory cells was evaluated on resting cells on D0. Activated cells were analyzed for viability, CD69 expression (activation) and proportion of naïve and memory cells. The figure shows one representative sample.


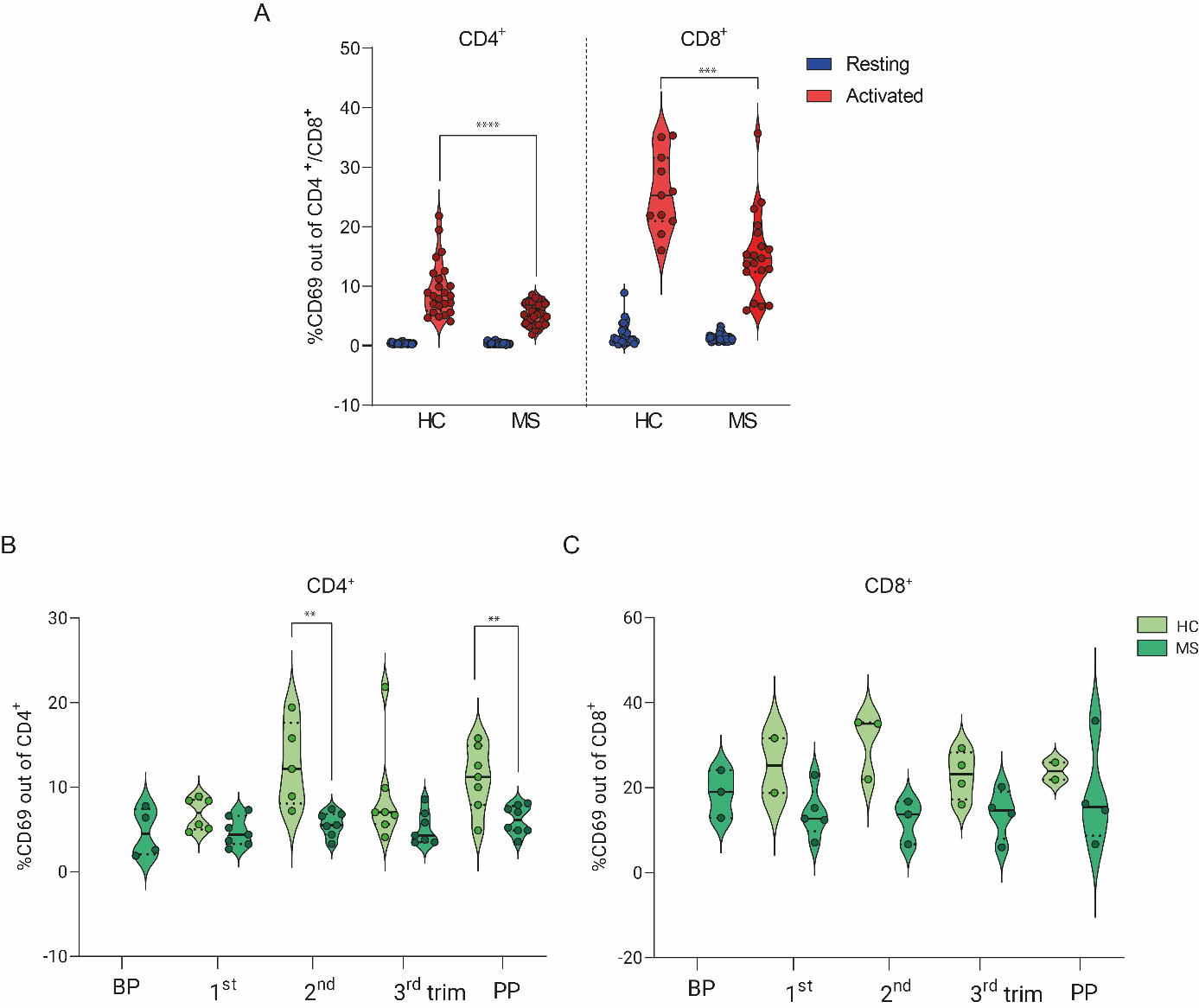


**Figure S14.** CD69 expression in resting and activated CD4^+^ and CD8^+^ T cells. **(A)** Proportion of CD69^+^ cells among CD4^+^ or CD8^+^ resting and activated T cells combining all time points within women with MS and healthy controls (HC). **(B,C)** CD69 expression in activated CD4^+^ and CD8^+^ cells before pregnancy (BP), 1^st^, 2^nd^ and 3^rd^ trimester and post-partum (PP). No activated cells were available before pregnancy for the healthy controls. The number of activated samples differ from the number of resting samples as not all samples had enough material to perform the T-cell activation assay, resulting in fewer activated samples (See materials and methods, section RNA sequencing for more details). Statistical differences were determined using an unpaired t test between MS and HC within each time point respectively. BP, before pregnancy; HC, healthy controls; MS, Multiple sclerosis; PP, post-partum; trim, trimester. **p<0.01, ***p<0.001, ****p<0.0001.


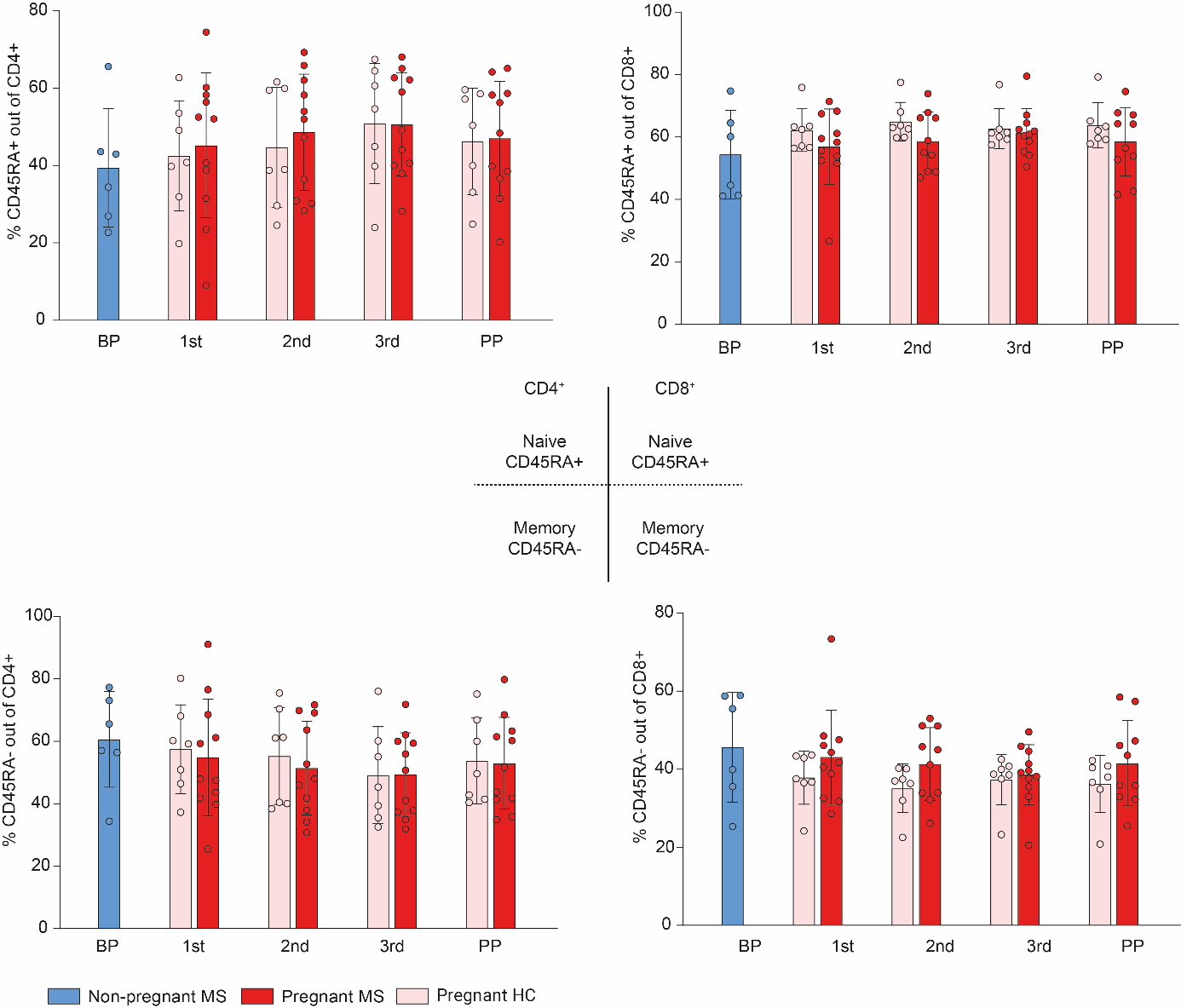


**Figure S15.** Proportions of naïve (CD45RA^+^) and memory (CD45RA^-^) in resting CD4^+^ and CD8^+^ cells from women with MS and healthy controls (HC) before, during and after pregnancy. MS and HC were also combined to compare differences over time irrespective of disease (dark red bar). There were no statistical differences within each group over time, between groups over time or at the same time point. Statistical differences were determined using one-way ANOVA. No statistically significant differences were found. BP; before pregnancy, HC, healthy controls; MS, Multiple sclerosis, PP; post-partum.
